# Omics-Based Mechanistic Insight Into the Role of Bioengineered Nanoparticles for Biotic Stress Amelioration by Modulating Plant Metabolic Pathways

**DOI:** 10.3389/fbioe.2020.00242

**Published:** 2020-04-17

**Authors:** Madhuree Kumari, Shipra Pandey, Shashank Kumar Mishra, Ved Prakash Giri, Lalit Agarwal, Sanjay Dwivedi, Alok Kumar Pandey, Chandra Shekhar Nautiyal, Aradhana Mishra

**Affiliations:** ^1^CSIR-National Botanical Research Institute, Lucknow, India; ^2^Academy of Scientific and Innovative Research, Ghaziabad, India; ^3^Department of Botany, Lucknow University, Lucknow, India; ^4^Department of Agriculture and Allied Sciences, Doon Business School, Dehradun, India; ^5^CSIR-Indian Institute of Toxicology Research, Lucknow, India

**Keywords:** nanoparticles, pesticide, plant pathogen, proteomics, metabolomics

## Abstract

Bioengineered silver nanoparticles can emerge as a facile approach to combat plant pathogen, reducing the use of pesticides in an eco-friendly manner. The plants’ response during tripartite interaction of plant, pathogen, and nanoparticles remains largely unknown. This study demonstrated the use of bioengineered silver nanoparticles in combating black spot disease caused by necrotrophic fungus *Alternaria brassicicola* in *Arabidopsis thaliana* via foliar spray. The particles reduced disease severity by 70–80% at 5 μg/ml without showing phytotoxicity. It elicited plant immunity by a significant reduction in reactive oxygen species (ROS), decreases in stress enzymes by 0.6–19.8-fold, and emergence of autophagy. Comparative plant proteomics revealed 599 proteins expressed during the interaction, where 117 differential proteins were identified. Among different categories, proteins involved in bioenergy and metabolism were most abundant (44%), followed by proteins involved in plant defense (20%). Metabolic profiling by gas chromatography–mass spectroscopy yielded 39 metabolite derivatives in non-polar fraction and 25 in the polar fraction of plant extracts. It was observed that proteins involved in protein biogenesis and early plant defense were overexpressed to produce abundant antimicrobial metabolites and minimize ROS production. Bioengineered silver nanoparticles performed dual functions to combat pathogen attack by killing plant pathogen and eliciting immunity by altering plant defense proteome and metabolome.

## Graphical Abstract

GRAPHICAL ABSTRACT

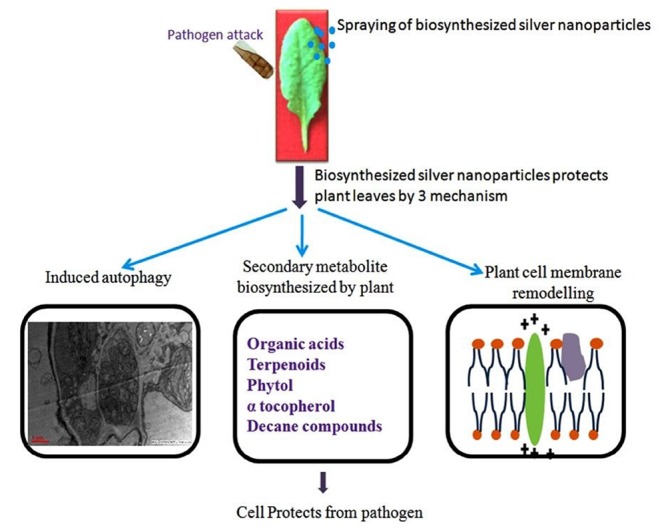

## Introduction

Plant diseases possess a potential threat toward the productivity and quality of food and fiber, putting agriculture on a higher pressure to feed the ever-increasing population. Among plants, the worldwide loss due to plant pathogens ranged from 50% in wheat to more than 80% in cotton (Oerke, 2006; Velásquez et al., 2018). Several management practices have been employed to overcome this problem, but the phenomenon of multidrug resistance acquired by pathogen and climate change has made the situation more difficult (Juroszek and Tiedemann, 2015; Kim et al., 2016).

Silver nanoparticles are potent antimicrobial agents, having proved their utility in medical applications (Mariana and De, 2019; Monir et al., 2019), however, their application in plant disease management is still in nascent stage (Ocsoy et al., 2013; Mishra et al., 2014). Stephano-Hornedo et al. (2020) and successfully controlled huanglongbing disease in Mexican limes (*Citrus aurantifolia* Swingle) by application of commercially available silver nanoparticles, found to be 3–60 times more efficient than conventional β-lactam antibiotics. Unlike chemical antimicrobial agents, silver nanoparticles exhibit multiple modes of actions of killing, which includes DNA unwinding, membrane disruption, changing permeability of membrane, and production of free radicals, making it very difficult for pathogens to acquire resistance against them (Verano-Braga et al., 2014; Kumari et al., 2017a, b).

Biogenic silver nanoparticles synthesized from biocontrol agent *Trichoderma viride* have proved their enhanced antimicrobial activity than their chemical counterparts by increased internalization inside cell in our previous studies (Kumari et al., 2017a). Recently, Mahawar et al. (2020) have also postulated a synergistic antifungal behavior of silver nanoparticles augmented with *Calothrixelenkinii* for controlling Alternaria blight disease by increase in polyphenol oxidase activity. Earlier, Ramalingam et al. (2016) had also observed higher ROS generation and increased changes in nano-mechanical properties of microbial cell surface after treatment with biogenic silver nanoparticles in comparison to chemically synthesized silver nanoparticles. Silver nanoparticles synthesized from chitin-induced exometabolites from a Trichoderma fusant were able to restrain the phytopathogen *Sclerotium rolfsii* at a minimum inhibitory concentration (MIC) of 20 μg/ml (Hirpara and Gajera, 2020) by modulating pathogenic gene expression. The effective role of silver nanoparticles in plant disease management *in vitro* and glass house conditions has been elucidated by some researchers, however, responses of plants toward the particles in fighting diseases remain unknown (Ocsoy et al., 2013; Ilaria et al., 2019).

An interaction between pathogen and plant follows a cascade of mechanism relying on the activation of the plant defense system and generation of reactive oxygen species (ROS), antioxidants, and stress enzymes (Gill and Tuteja, 2010). Several inducers and strategies have been employed to improve plant resistance toward pathogens (Ahuja et al., 2012; Hassan and Mathesius, 2012). Defense activities are elicited after attempted microbial infection including the deposition of the phenolics, ascorbic acid, and induction of antioxidant enzymes to overcome the stress (Singh et al., 2013).

A detailed investigation to decipher the changes occurring on the molecular level of the plant system during the tripartite interaction of plant, pathogen, and nanoparticles is needed. Development in the area of “proteomics” has opened new opportunities to explore large-scale data-rich biology of plants at protein levels. Mass spectrometry (MS)-based comparative proteomics can be a useful tool to determine the physiological status of plants (Verano-Braga et al., 2014). Metabolomics occupies a significant position among several omics approaches to know the physiological status of a cell, comprising the non-genetically encoded substrates, intermediates, and products of metabolic pathways, associated to a cell (Nielsen and Jewett, 2007; Chen et al., 2020).

In this study, the model pathosystem of *Arabidopsis thaliana* ecotype Columbia (col-0) and *Alternaria brassicicola* was investigated to assess the potential of biogenic silver nanoparticles in plant disease management. *A. brassicicola* causes black spot disease on virtually every important cultivated *Brassica* species. Proteomics, metabolomics, defense, and morphological response of plant during the tripartite interaction of plant, pathogen, and nanoparticles were also evaluated to get an insight into the mechanistic aspects of this interaction.

## Materials and Methods

### Nanoparticles

Spherical biogenic silver nanoparticles (2–5 nm) (SNP) were synthesized earlier from cell-free extract of *T. viride* (MTCC 5661) at specified conditions (Kumari et al., 2017b, 2019) and concentration was adjusted to 5 μg/ml before use. Particles were filtered through 0.22 μm syringe millipore filters and sonicated for 2 min before use for getting monodispersed population. Zeta size and zeta potential of nanoparticles were measured following the protocols of Kumari et al. (2017b) (Supplementary Figure S1).

### Detached Leaf Assay

Detached leaf assay was performed with different concentrations of SNP (1, 2.5, 5, and 7.5 μg/ml). Briefly, 1-month-old *A. thaliana* leaves were washed with ultrapure water and surface sterilized by 0.4% sodium hypochlorite solution for 30 s, followed by rinsing with sterile water five times. Leaves were dipped in 5 ml of nanoparticles for 30 min and placed on 0.8% agar plates while leaves dipped in sterile water served as control. Spore suspension (10^5^ spores/ml) of *A. brassicicola* was prepared in 1% gelatin and spotted on leaves (2 spots/leaf) with 10 μl of spore suspension. Reduction in disease severity was calculated by measuring the reduction in spore count as compared to control leaves.

### Plant *in vivo* Experiments

Seeds of *A. thaliana* ecotype Columbia (Col-0) were surface sterilized by 0.4% sodium hypochlorite solution followed by repeated washing with distilled water. Surface-sterilized seeds were sown (10 seeds) on sterile soilrite and pots were transferred to temperature-controlled culture room (set at 22°C) in continuous light conditions after 3 days of cold treatment (Srivastava et al., 2012b). They were irrigated twice a week with the nutrient solution and the plants were allowed to grow for 1 month after germination. After 1 month, pots were divided into four groups with six replicates each:

1. Control (Con);
2. Plants treated with biogenic silver nanoparticles (SNP alone);
3. Plants infected with *A. brassicicola* (AB alone);
4. Plants with both silver nanoparticles and challenged with *A. brassicicola* (AB + SNP).

Control plants were sprayed with 1% gelatin, while SNP and AB + SNP treatments were sprayed with the biogenic nanoparticles of concentration 5 μg/ml until runoff. Two hours after treatment, AB and AB + SNP treatments were inoculated with spore suspension of *A. brassicicola* (10^5^ spores/ml). Plants were bagged with a transparent plastic bag and kept for 48 h. After 48 h of infection, leaf samples were harvested immediately in liquid nitrogen and kept in -80°C till further analysis for biochemical and omics analysis. For staining, fresh leaves were collected and proceeded further with the mentioned protocols. Disease parameters and morphological data, viz., number of lesions/leaf, number of spores/leaf, chlorophyll content, and fresh and dry weight of leaves were also taken from fresh leaves 48 h post-infection.

### Biochemical Analysis

Total chlorophyll content in plants was determined by the protocol given by Arnon (1949). Defense parameters of plants including proline, lipid peroxidation (LPX), hydrogen peroxide (H_2_O_2_), ascorbic acid, and total phenolics were measured following the protocols of Bates et al. (1973); Heath and Packer (1968), Sergiev et al. (1997); Mitusi and Ohata (1961), and Mallick and Singh (1980), respectively, with some modifications. Antioxidant enzymes, superoxide dismutase (SOD), catalase, GoPX, phenylalanine ammonia lyase (PAL), and polyphenol oxidase (PPO) were measured following the protocols of Beauchamp and Fridovich (1971); Aebi (1974), Hemeda and Klein (1990) and Lavania et al. (2006), respectively. All analyses were carried out on UV-vis spectroscopy (Perkin Elmer, MA, United States), keeping length of light path 1 cm.

### Histochemical Staining

After 48 h of infection, leaves were preserved in acetic acid:ethanol (1:3) and stained with 0.1% trypan blue for fungal colonization and 0.1% lactophenol cotton blue for mycelia growth inhibition. For observation of the slides, light microscope (Leica Microsystems, GmbH, Germany) was used at CSIR-NBRI, Lucknow.

### Scanning and Transmission Electron Microscopy

For evaluation of morphological differences between the treatments, transmission electron microscopy (TEM) and scanning electron microscopy (SEM) were carried out with some modification of protocols (Tiwari et al., 2014) at the central instrument facility of CSIR–Indian Institute of Toxicology Research (IITR), Lucknow.

After 48 h of infection, fresh leaves of *A. thaliana* were cut and fixed in 4% formaldehyde and 2% glutaraldehyde in cacodylate buffer 0.1 M, pH 6.9. After 3 days, the samples were rinsed in 0.1 M cacodylate buffer and post-fixed in 1% osmium tetroxide in cacodylate buffer, pH 6.9, for 2 h at 4°C. The pieces were then rehydrated in propylene oxide, embedded in an araldite and dodecenyl succinic anhydride mixture, and baked for 48 h at 65°C. Ultrathin sections (5070 nm) were cut with a Leica EMUC7 ultramicrotome, stained with uranyl acetate and lead citrate, and examined under TEM (FEI, TechnaiG2 Spirit TWIN, United States).

For SEM studies, fixed and dehydrated samples were dried by critical point method. The samples were spur coated by gold metal by sputter coater and were analyzed in high vacuum mode by high-resolution field emission E.M., Quanta SEM field emission gun (FEG 450, Netherlands).

### Proteomic Profiling

#### Protein Extraction

Proteins were isolated from leaves of *A. thaliana* from three replications to normalize the effect of biological variations. In brief, samples were ground in liquid N_2_, and the resulting powder was extracted with lysis buffer containing 50 mM Tris–HCl, pH 7.5, 60 mM KCl, 10 mM MgCl_2_, 0.5% Triton X, 2 mM PMSF, and 1 mM DTT. The extract was incubated overnight at 4°C on a rotary shaker at 180 rpm and further centrifuged at 15,000 rpm (25,200 *g*) for 15 min at 4°C. The extracted protein was precipitated with 0.1 M ammonium acetate (in 100% methanol) at -20°C and then centrifuged at 15,000 rpm for 15 min at 4°C. The pellet was washed thrice with chilled acetone. Further, it was dried and resuspended in a solubilization (rehydration) buffer consisting of 8 M urea, 2 M thiourea, 4% CHAPS, and 20 mM DTT and quantified by Bradford method (Agarwal et al., 2016).

#### Two-Dimensional Gel Electrophoresis, Gel Staining, and Protein Identification

Two-dimensional electrophoresis (2-DE) was carried out following the protocols of Agarwal et al. (2016) with some modifications [details provided in Supporting Information (SI)].

#### MS and MS/MS

A 4800 Proteomics Analyzer (Applied Biosystems, MA, United States) with TOF/TOF optics was used for all MALDI-MS and MS/MS applications. Samples were prepared by mixing 0.5 ml of sample with 0.5 ml of matrix solution (5 mg/ml a-Cyano-4-hydroxycinnamic acid in 50% ACN containing 0.1% TFA) and spotted on stainless steel 384-well target plate. They were air dried at room temperature and were then placed in the mass spectrometer and subjected to mass analysis. The mass spectrometer is externally calibrated with a mixture of angiotensin I, Glufibrino–peptide B, ACTH (1e17), and ACTH (18e39). For MS/MS experiments, the instrument was externally calibrated with a fragment of Glufibrino–peptide B. The monoisotopic peptide masses obtained from MALDI-TOF-TOF were analyzed by the 4000 Series Explorer software version 3.5 (ABI, MA, United States). Based on mass signals, protein identification was carried out with the Mascot software^1^ to search proteins against NCBInr databases. The parameters used for database searches were as follows: monoisotopic mass accuracy, <100 ppm; missed cleavages, 1; carbamidomethylation of cysteine as fixed modification and oxidation of methionine, and N-terminal acetylation (protein) as variable modifications. The only protein spots whose MOWSE score was above the significant threshold level determined by Mascot was considered to represent an identification. The protein functions were assigned using the Pfam^2^ and InterPro^3^ protein function databases. Self-organizing tree algorithm (SOTA) clustering was performed on the log-transformed fold induction expression values across different treatments using the Multi Experiment Viewer (MEV, 4.9) software. The clustering was done with the Pearson correlation as distance with 10 cycles and a maximum cell diversity of 0.8. Increase or decrease at least in one treatment was considered for differential spot identification. A criterion of *p* < 0.001 was used to determine the significant difference for analyzing the parallel spots between genotype with analysis of one-way variance (ANOVA).

### Metabolic Profiling

#### Extraction of Metabolites

The lyophilized plant material (1.0 g) was extracted with hexane (35°C) in a ratio of 1:10 w/v. The solvent was separated by filtration and the procedure was repeated until the hexane layer became colorless. Thus, the separated solvent layer was concentrated under reduced pressure and the obtained sticky mass was stored at -20°C for further GC–MS analysis. The remaining solid plant material was further extracted thrice with fivefold excess (w/v) of 90% and then with 70% methanol–water. The extract obtained was reduced using rotavapor and defatted with the equal volume of hexane and further concentrated under reduced pressure. The samples were then lyophilized to dryness and the resulting solid was again stored at -80°C for further analyses (Srivastava et al., 2012a).

#### GC-MS Analysis

GC-MS analysis was performed following the protocols of Srivastava et al. (2012a) using Thermo Trace GC Ultra coupled with Thermo Fisher DSQ II mass spectrometers with electron impact ionization at 70 eV to generate mass spectra (details provided in Supplementary Material).

#### Quantification of Silver Ions in Plant Leaves

Quantification of silver ions present in leaves of the plant was done by the protocols of Srivastava et al. (2018) by inductively coupled plasma mass spectrometer (ICP-MS, Agilent 7500 cx). Briefly, 500 mg of dried *Arabidopsis* leaves were digested with 5:1 v/v of HNO_3_ and H_2_O_2_ at 120°C for 4 h. Total silver was quantified in the digested sample and analyzed by the ICP-MS. Silver was used as a standard and for calibration (300 ppb, Agilent).

### Statistical Analysis

The results were expressed as the mean values ± SE (standard error of mean) of mg/g for non-polar compounds and μg/g for polar compound obtained in GC-MS analysis. The statistical significance for the four different treatments was determined by one-way ANOVA *post hoc* Bonferroni multiple comparison test (SPSS 11.5.0, United States) with the probability of *P-*value of ≤ 0.05.

## Results and Discussion

### Detached Leaf Assay

The spore count reduced to 32.66, 61.66, 85.86, and 86% in silver nanoparticle-treated *A. brassicicola*-infected plants (AB + SNP) when treated with 1, 2.5, 5, and 7.5 μg/ml of SNP, respectively, in comparison to *A. brassicicola*-infected plants (AB alone) (Supplementary Figure S2). Detached leaf assay is one of the easy methods that mimic the plants’ responses during stress conditions and *in vitro* conditions.

Based on the results obtained, 5 μg/ml of SNP was selected for further studies.

### Evaluation of Biosynthesized Silver Nanoparticles on Pathogenesis of *A. brassicicola* on Model Plant *A. thaliana*

**FIGURE 1:**
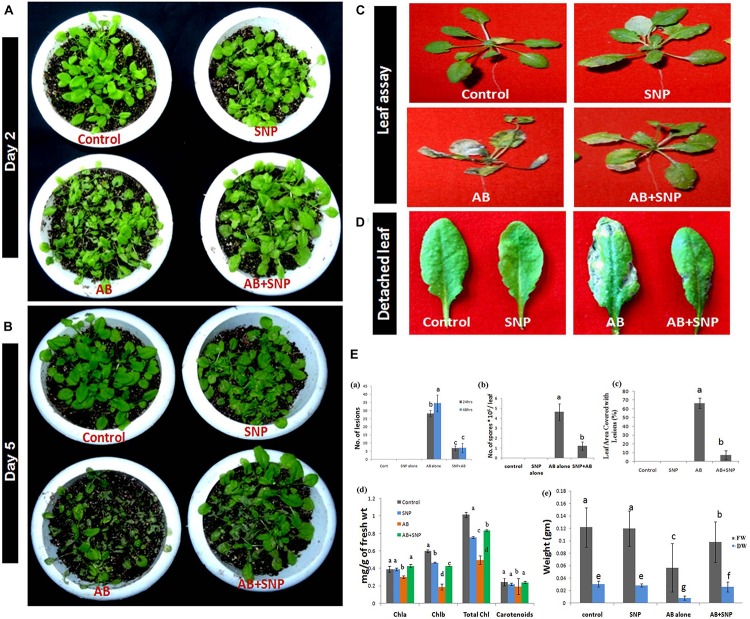
Photograph showing effect of silver nanoparticles in reducing disease severity after **(A)** 48 h (day 2) post infection **(B)** Day 5 post infection **(C)** reduction in necrosis of leaves **(D)** reduction in number of lesions formed per leaf **(E)** Assessment of disease parameters in terms of **(a)** number of lesions **(b)** number of spores **(c)** leaf area covered with lesion **(d)** chlorophyll content **(e)** fresh and dry weight in silver nanoparticles pre-treated plants as compared to other treatments. Cont-Control, SNP-Biogenic silver nanoparticles lone, AB-*A. brassicicola* infected plants, AB + SNP-*A. brassicicola* infected, treated with SNP, FW-fresh weight, DW-Dry weight. Values are the means ± SD of three replicates. Means sharing different alphabets “a”, “b” differ significantly from each other at *p* ≤ 0.05.

From visual observation, results similar to detached leaf assay were obtained indicating potency of SNPs as antifungal agents (Figure 1). After 48 h of infection, lesions and necrosis were observed in parts of the plant in AB alone (Su’udi et al., 2013), however, SNP pre-treated leaves were as healthy as control (Figure 1A). Leaf area covered with lesions in AB + SNP decreased significantly in comparison to AB alone after 48 h of infection (Figures 1A,E). After 5 days of infection, AB-alone plants showed severely damaged leaves, while AB + SNP plants controlled the visual symptoms of the disease (Figure 1B). There was a significant reduction of lesions (80%) and spores (74.13%) after 48 h of infection in AB + SNP as compared to AB alone (Figures 1C–E). Number of spores, lesions in leaf, and leaf area covered with lesions are important parameters to assess the disease severity in plants (Yan et al., 2002). This study corroborated well with the previous studies reporting biosynthesized silver nanoparticles as an efficient means to control plant diseases (Anandaradje et al., 2020; Shen et al., 2020). Chlorophyll content in AB only reduced by 51.2%, while the reduction in fresh and dry weight was 58.33 and 73.3%, respectively, as compared to control (Figures 1Ed,e). AB + SNP exhibited 68.68, 96, and 212.5% increase in chlorophyll content and fresh and dry weight, respectively, as compared to AB alone (Figures 1Ed,e). Loss of photosynthetic pigments is one of the major biochemical changes a plant undergoes during *Alternaria* infection (Meena et al., 2016). Increase in chlorophyll content in AB + SNP in comparison to AB alone showed the increased disease resistance of plant toward disease after treatment with SNPs. Further, no phytotoxicity of SNP was observed in terms of fresh and dry weight and chlorophyll content in plants (Figures 1Ed,e). Lactophenol cotton blue-stained leaves demonstrated a network of mycelia in AB only, which was not observed in AB + SNP (Supplementary Figures S3A,B). Lactophenol cotton blue is a stain used to distinguish fungal mycelia from plant cells, which stains fungal hyphae in blue color, leaving plant cell wall unstained (Marques et al., 2013). Similarly, trypan blue is another stain used to distinguish between dead and live tissues (Fernández-Bautista et al., 2016). Trypan blue-stained leaves showed higher viability in AB + SNP as compared to AB alone (Supplementary Figures S3C,D). The larger necrotic lesions formed after Alternaria infection in AB stained more profoundly in comparison to the smaller and lesser lesions in AB + SNP.

### Scanning Electron Microscopy

**FIGURE 2:**
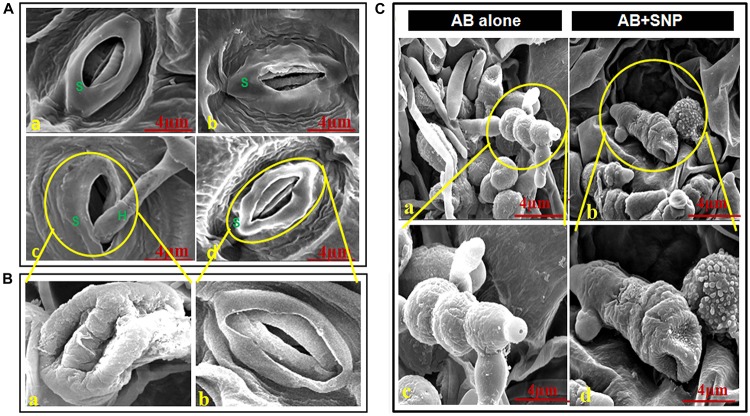
Scanning electron micrographs of adaxial surface of *A. thaliana* leaf. **(A)** Stomata of **(a)** control, **(b)** SNP, **(c)** AB alone, and **(d)** AB + SNP. S represents stomata and H represents hyphae of fungal pathogen. **(Ba)** Enlarged image of damaged stomata in AB alone and **(b)** enlarged image of normal stomata in AB + SNP treatment. **(Ca,c)** Image showing healthy mycelia and spore network on plant surface in AB alone. **(b,d)** Absence of mycelia network and damaged spores in AB + SNP. Cont, control; SNP, biogenic silver nanoparticles alone; AB, *A. brassicicola*-infected plants; AB + SNP, *A. brassicicola*-infected, treated with SNP.

Adaxial surface of *A. thaliana* leaves with SNP and AB + SNP treatment showed no alteration in surface morphology (Figures 2A,b,d), however, AB-only plant showed healthy mycelia network over leaf surface (Figures 2C,a,c).

The images also demonstrated the hyphae entering into the cell through stomata and consequent damage (Figures 2B,a). The AB + SNP treatment did not only inhibit the conidial germination, but also created structural aberrations in fungal conidia (Figure 2C). The spores of *A. brassicicola* in AB + SNP treatment were destroyed with severe deformation. Ocsoy et al. (2013) had observed similar deformations in tomato pathogen *X. perforans* by DNA-directed silver nanoparticles on graphene oxide. SNPs show multiple modes of actions including cell wall and cell membrane damage, denaturation of protein, and DNA unwinding to kill the pathogen (Ramalingam et al., 2016).

### Determination of Antioxidant Activities of *A. thaliana*

#### Non-enzymatic Parameters

Phenolic compounds are the key of plant immune response, which participates in activating plant defense genes, production of phytoanticipins and phytoalexins, structural barriers, and modulation of pathogenicity (Shoresh and Harman, 2008). There was an increase of 47.82% in total phenolics in AB + SNP, as compared to AB-only plants (Supplementary Figure S4A). Krishnaraj et al. (2012) had demonstrated the elevated level of phenolics in the SNP-treated plants and speculated that mild stress might be beneficial to plants in protecting them against pathogen attacks. Content of ascorbic acid was highest in AB-only plants followed by AB + SNP and SNP treatment (Supplementary Figure S4B). Ascorbic acid is a well-known antioxidant and precursor of many defense molecules (Abdelrahmana et al., 2016). Higher content of ascorbic acid in pathogen-infected plants may be due to the higher requirement of antioxidants to combat biotic stress.

Content of thiobarbituric acid reactive substances (TBARS) and proline increased 55.3 and 368.3%, respectively, in AB-only plants, whereas the AB + SNP plants showed 28.47% reduction in TBARS content and 221.7% reduction in proline as compared to AB alone (Supplementary Figures S4C,D). TBARS content depicts the peroxidation of lipid membrane caused by ROS species (Singh et al., 2013). Reduction in TBARS content of AB + SNP indicated the healthier status of plants. Similarly, proline increases during the stress condition, which acts as a signaling molecule to modulate mitochondrial functions, influence cell proliferation or cell death, and trigger specific gene expression (Szabados and Savoure, 2009).

Further, to assess the ROS generation, H_2_O_2_ production was quantified. It was found that H_2_O_2_ level reduced to 28% in AB + SNP as compared to AB-only plants (Supplementary Figure S4E). There was slight increase in proline, TBARS, and H_2_O_2_ content in SNP alone, which might be an indicator of inducing plant immune response prior to pathogen infection.

#### Stress Enzyme Activity

**FIGURE 3:**
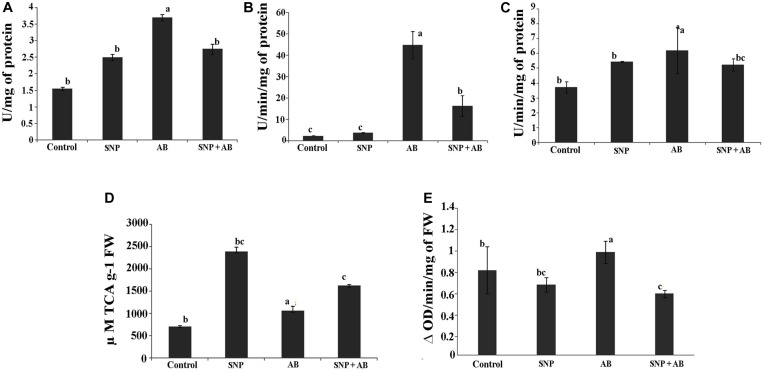
Stress enzyme activities across all the treatments in terms of **(A)** SOD, **(B)** catalase, **(C)** GoPX, **(D)** PAL, and **(E)** PPO. Cont, control; SNP, biogenic silver nanoparticles alone; AB, *A. brassicicola*-infected plants; AB + SNP, *A. brassicicola*-infected, treated with SNP. Values are the means ± SD of three replicates. Means sharing different letters “a” and “b” differ significantly from each other at *p* ≤ 0.05.

Among the four treatments, SOD, CAT, and GoPx were maximum in AB alone. SOD, CAT, and GoPx activities were enhanced by 1. 6-, 19. 8-, and 0.6-fold in AB alone while a decrease in stress enzymes was observed in AB + SNP plants (Figures 3A–C).

Infection of *A. brassicicola* induced the generation of ROS, thereby increasing the enzymatic activity to scavenge the ROS generated (Gill and Tuteja, 2010) in AB alone. The AB + SNP plants were able to combat the pathogen infection due to the antagonistic effects of SNP on *A. brassicicola* spores and activated systemic resistance of *A. thaliana.* Further, to study the effect of tripartite interaction of plant, pathogen, and nanoparticles on phenylpropanoid pathway, which stimulates disease resistance factors including phytoallexins and phenolics (Lavania et al., 2006), PAL and PPO activities were assessed. PAL activity was highest in the plants pre-treated with silver nanoparticles (SNP and AB + SNP) (Figures 3D,E), resulting in production of phenolic compounds (Supplementary Figure S4A), many of which are antimicrobial and can show a synergistic effect with silver nanoparticles to ameliorate the biotic stress. PPO activity was highest in the AB plants, followed by AB + SNP and SNP as compared to control (Figure 3E). The production of phenolic compounds before infection, due to the presence of SNP, might be a reason for strengthened immunity of plant to reduce the generation of ROS. Further, to gain an insight into the molecular responses of plants during the interaction, proteomics and metabolomics were carried out.

### Transmission Electron Microscopy

**FIGURE 4:**
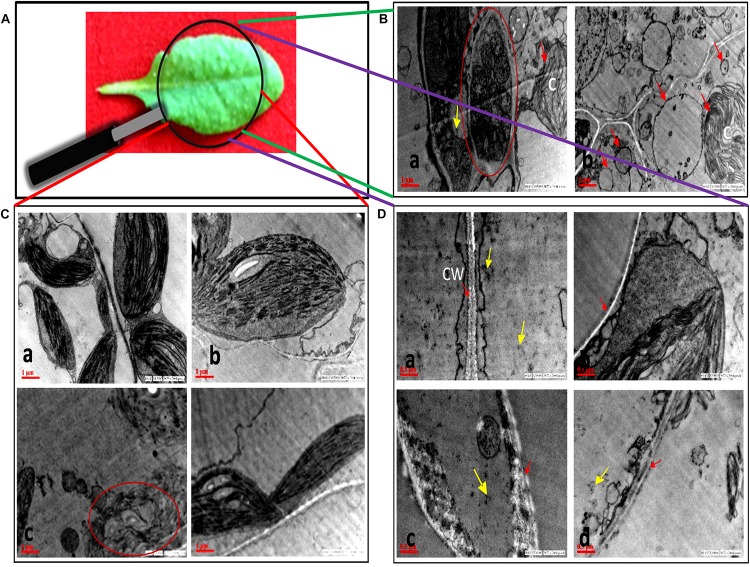
Transmission electron micrographs of cell organelles of *A. thaliana* leaves across all treatments. **(A)** Sample image of *A. thaliana* leaf. **(B)** Autophagosomal structures in AB + SNP treatments. **(a)** Single membrane vesicles encapsulating several organelles (encircled with red) near chloroplast. **(b)** Membrane-bound autophagosomal structure near chloroplast. Arrow represents single-membrane autophagosomal structure, chloroplast is represented by **(C)**. **(C)** Chloroplast of **(a)** control, **(b)** SNP, **(c)** AB alone, and **(d)** AB + SNP. Encircled image of AB represents damage in chloroplast. **(D)** Cell wall of **(a)** control, **(b)** SNP, **(c)** AB alone, and **(d)** AB + SNP. Arrow represents cell wall (CW). Cont, control; SNP, biogenic silver nanoparticles alone; AB, *A. brassicicola*-infected plants; AB + SNP, *A. brassicicola*-infected, treated with SNP. Yellow arrows represents SNPs.

No morphological changes were observed in SNP-treated leaves as compared to control (Figure 4), while damage was observed in chloroplast and cell wall of AB alone (Figures 4B,Cc). The symptom reflected the infection of *A. brassicicola* and secretion of toxins (Otani et al., 1995). In AB alone, the internal lamellar system was retained but thylakoids were not as compactly arranged as in control. Damage in the cell wall of AB-alone plants was visible by scattered white deposition of cellulose, which was absent in AB + SNP treatment. The most interesting finding of this study was the observation of autophagic granules in AB + SNP treatment (Figure 4B). Autophagy has emerged as a powerful weapon of plants against necrotrophic pathogens (Kabbage et al., 2013). Single-membrane vesicles encapsulating several organelles (Figure 4Ba), depicting early lysosomal activities (Figure 4Bb), were observed near chlorophyll in AB + SNP treatment. In both figures, the activity was observed adjacent to chloroplast and demonstrates chlorophagy, which can be a mechanism to lower down ROS production and enhance plant immunity (Dong and Chen, 2013).

### Proteome Regulation by Biosynthesized Silver Nanoparticles

To study the role of proteins associated with resistance in *Arabidopsis* plant against *A. brassicicola*, comparative proteomics analysis was carried out from control, SNP alone, AB alone, and AB + SNP treatments.

For each of the treatments, three replicates gels were run and images were analyzed by the PDQuest software version 8.0.1. The mean value of the high-quality spots was used as the spot quantity on the master gel (Supplementary Figure S5 and Supplementary Table S1).

**FIGURE 5:**
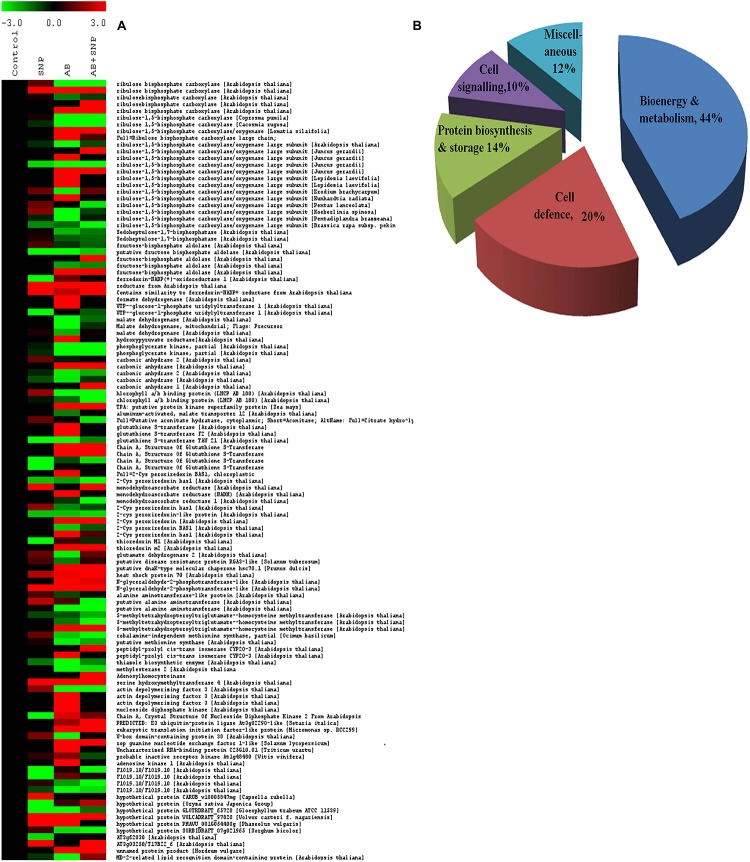
**(A)** Clustering of differentially expressed proteins. Each protein is represented by a single row of colored boxes. Induction to suppression ranges from red to green. **(B)** Functional cataloging of differentially expressed proteins. The identified proteins were assigned a putative function using protein function databases and functionally grouped in five different categories as represented in the pie chart. Cont, control; SNP, biogenic silver nanoparticles alone; AB, *A. brassicicola*- infected plants; AB + SNP, *A. brassicicola*-infected, treated with SNP.

A total of 599 high-quality spots were detected in master gel image in which 144 spots were found to be differentially expressed by more than 2.5-fold. The differential spots obtained were further subjected to MALDI MS/MS in which 117 proteins were identified with the significant score (Supplementary Table S2). Out of 117 identified, 50 proteins were upregulated, 33 were downregulated, and 34 demonstrated a mixed pattern of differential protein expression among four treatments (Figure 5A). A total of 63 proteins were unique in identification, while the rest were the result of post-translation or members of multigene families (Agrawal et al., 2013).

The details of the MS/MS analyses, including protein identification, the score of the identified protein, threshold score, number of matched peptides, and % sequence coverage, are listed in Supplementary Table S3. Result analysis showed that most of the protein spots quantitatively differ in different treatment along with qualitative difference. Supplementary Figure S6 represents some typical gel regions showing protein spots in the enlarged dotted box of Supplementary Figure S5 with quantitatively and qualitatively altered expression.

#### Differentially Expressed Proteins Based on Functional Classes

To get an insight into the differentially expressed proteins, identified proteins were further divided into five functional classes based on their putative function, *viz*., bioenergy and metabolism (BEM) with highest number of protein (44%), plant defense (20%), cell signaling (14%), storage and biogenesis (10%), and miscellaneous (12%) (Figure 5B). Detailed information regarding differentially expressed proteins has been provided in Supplementary Material.

#### Hierarchical Clustering of Differentially Expressed Proteins

**FIGURE 6:**
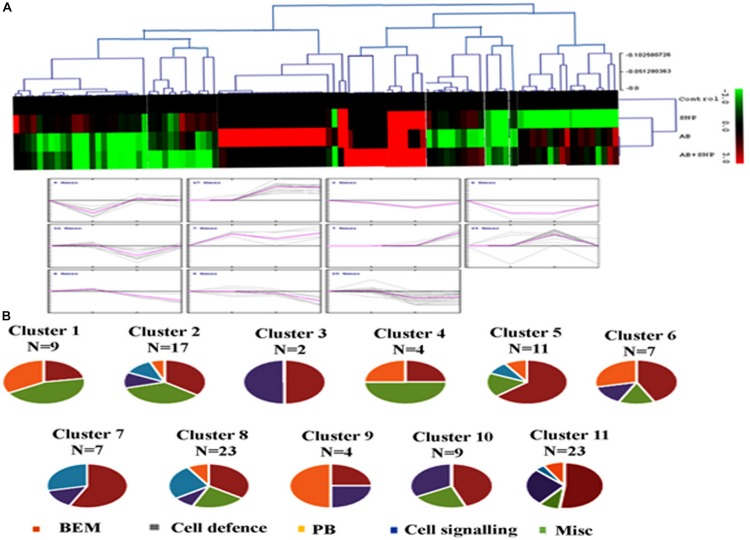
Clusterogram of differentially expressed proteins showing 11 clusters based on their expression profiles. **(A)** The SOTA cluster trees and **(B)** expression profiles of 11 different clusters. Pie chart represents respective contribution of proteins of different functional groups. Cont, control; SNP, biogenic silver nanoparticles alone; AB, *A. brassicicola*-infected plants; AB + SNP, *A. brassicicola*-infected, treated with SNP.

To know the expression pattern of different proteins, and group them accordingly, unbiased clustering was performed using the SOTA algorithm. Proteins were grouped according to the pattern of their expression, using the Pearson correlation coefficient as the distance function. The data used in the study was fold change in expression of proteins with respect to the control protein expression value. The analysis yielded 11 different clusters of proteins, representing a similar expression pattern for each cluster (Figures 6A,B), however, only clusters having *n* ≥ 5 were taken into consideration for further analysis.

Cluster 1 was represented by nine proteins, dominated by the proteins related to plant defense and stress. As observed earlier, the maximum expression was found in pathogen-infected plants while proteins were downregulated in the treatment of silver nanoparticles. Cluster 2 represented 17 proteins in which most of them were involved in BEM and plant defense and rescue. This cluster also showed upregulation in infected plants, while the expression pattern of SNP was similar to control. Clusters 5, 6, 10, and 11 were also dominated by proteins involved in BEM, downregulated in infected plants, and upregulated in SNP and AB + SNP treatment as compared to AB alone. Cluster 7 had proteins involved in cell signaling, which were highly upregulated in AB + SNP treatment. Apart from proteins involved in BEM, clusters 9 and 10 also showed proteins of protein biogenesis, showing upregulation in SNP as observed earlier in this study. Altogether, the hierarchical clustering suggests the different pattern of expression of all proteins, which, along with function classification, can help in elucidation of the mechanism involved behind nanoparticle-mediated plant protection against pathogenic fungi.

#### Domain Analysis

Further domain analysis was also carried out to determine the putative functions of unknown proteins (Supplementary Table S4). AtC 2024, 3015, 1030, 8431, and 8529 were found to have ribulose bisphosphate carboxylase small-chain domain, while AtC 4229 contained myosin S1 fragment, N-terminal domain. AtC 6230 was found to have S-adenosyl-L-methionine-dependent methyltransferase-like domain while AtC6619 had nucleotide-diphospho-sugar transferases domain. Interestingly, AtC 7415, 8411, and 4236 had ABC-transporter extracellular N-terminal domain, zinc finger CCHC-type domain, and cyclophilin-like domain, respectively. AtC 7415 and 8411 were upregulated in the plants with SNP treatment.

ABC transporters are one of the largest protein families involved in transport across cell membrane, performing a wide range of functions (Kang et al., 2011). Recently, their role in conferring plant resistance against pathogens and tolerance to abscisic acid has been elucidated (Ji et al., 2014). Similarly, cyclophilins are plant proteins involved in a variety of functions including development and stress responsiveness (Romano et al., 2004). Zinc finger CCHC-type domain-containing proteins comprise a large conserved protein family across the eukaryotic system and play a critical role in mRNA metabolism (Bogamuwa and Jang, 2014). Many of the zinc finger proteins are potential transcription factors that regulate plant responses toward biotic and abiotic stress (Nakai et al., 2013; Yang et al., 2014). Differential expression of proteins containing domains described above needs a detailed investigation to decipher the nanoparticle-mediated changes in plant proteome during plant–pathogen–nanoparticles interaction.

### Metabolic Profiling

#### GC-MS Analysis

A total of 39 metabolite derivatives in non-polar (CHCl_3_) fraction (Table 1) and 25 in polar (water) fraction (Table 2) of plant extracts with four treatments (Control, SNP, AB, and AB + SNP) were detected by GC-MS analysis. The polar metabolites included diverse groups of organic acids, sugars, and intermediates of Krebs cycle, while non-polar metabolites mainly consisted of organic acids, fatty acids, and sterols. GC-MS analysis allows the metabolites to separate on the basis of their mass/charge ratio, which together can serve as a tag for that metabolite (Srivastava et al., 2012a).

**TABLE 1:** Quantitative variability in non-polar metabolites of *A. thaliana* leaves during plant–pathogen–nanoparticles interaction.

**Rt (min)**	**Metabolites**	**Fragmentation patterns**	**Control (mg/g)**	**SNP (mg/g)**	**AB (mg/g)**	**SNP + AB (mg/g)**
6.79	Ethylene glycol	m/z 206,193,191,147,103,73	0.450 ± 0.047^a^	0.290 ± 0.024^c^	0.390 ± 0.041^ab^	0.350 ± 0.036^bc^
9.33	Propanoic acid	m/z 234,220,219,147,117,73,66,55	ND^a^	0.190 ± 0.020^b^	ND^a^	0.380 ± 0.040^b^
9.55	Caproic acid	m/z 188,174,131,75,73,69	ND^b^	ND^b^	ND^b^	0.040 ± 0.004^a^
16.01	Glycerol	m/z 308,293,219,205,148,117,75,73,55	ND^b^	ND^b^	ND^b^	0.960 ± 0.100^a^
18.09	Non-anoic acid	m/z 230,216,171,155,117,85,73,55	ND^b^	0.200 ± 0.021^a^	ND^b^	ND^b^
18.84	Cyclotetradecane	m/z 196,168,126,97,83,69,55,51	ND^b^	0.150 ± 0.016^a^	ND^b^	ND^b^
19.06	Tetradecane	m/z 198,127,113,99,71,57	ND^b^	3.270 ± 0.340^a^	0.110 ± 0.011^b^	ND^b^
21.59	Pentadecane	m/z 212,127,113,99,71,55,54	ND^c^	4.310 ± 0.449^a^	0.060 ± 0.006^c^	0.680 ± 0.071^b^
23.98	Hexadecane	m/z 226,127,113,99,85,71,57	ND^c^	7.810 ± 0.813^a^	ND^c^	2.600 ± 0.271^b^
25.24	Lauric acid	m/z 272,259,229,185,117,83,73,55	ND^c^	0.690 ± 0.072^a^	0.060 ± 0.006^c^	0.430 ± 0.045^b^
26.22	Heptadecane	m/z 240,169,127,113,99,85,71,55,53	ND^c^	4.590 ± 0.478^a^	ND^c^	1.890 ± 0.197^b^
26.35	Pristane	m/z 268,249,141,113,85,71,57,56	ND^b^	1.340 ± 0.139^a^	ND^b^	ND^b^
28.37	Octadecane	m/z 254,169,141,113,99,85,71,57	ND^c^	7.390 ± 0.769^a^	ND^c^	3.730 ± 0.388^b^
28.55	Phytane	m/z 282,249,183,155,127,85,71,55	ND^b^	0.770 ± 0.080^a^	ND^b^	ND^b^
29.19	Neophytadiene	m/z 278,263,195,138,95,68,57,55,51	9.800 ± 1.020^a^	ND^d^	3.970 ± 0.413^b^	1.790 ± 0.186^c^
29.42	Myristic acid	m/z 300,287,201,148,132,117,73,55	ND^d^	0.700 ± 0.073^c^	0.990 ± 0.103^b^	1.160 ± 0.121^a^
30.39	Non-adecane	m/z 268,197,155,127,113,99,85,71,57	ND^c^	4.140 ± 0.431^a^	ND^c^	2.160 ± 0.225^b^
31.39	n-Pentadecanoic acid	m/z 314,300,255,145,117,85,75,73,55	ND^b^	ND^b^	ND^b^	0.390 ± 0.041^a^
32.34	Eicosane	m/z 282,211,141,113,99,85,71,57	ND^c^	5.760 ± 0.600^a^	ND^c^	3.360 ± 0.350^b^
32.77	cis-5,8,11-Eicosatrienoic acid	m/z 378,348,232,129,117,75,73,55	0.390 ± 0.041^c^	0.580 ± 0.060^c^	1.290 ± 0.134^b^	1.640 ± 0.171^a^
34.19	Heneicosane	m/z 296,225,141,127,99,85,71,57	ND^c^	3.630 ± 0.378^a^	ND^c^	1.030 ± 0.107^b^
35.65	Phytol	m/z 368,353,213,157,143,123,75,73,57	0.870 ± 0.091^d^	1.490 ± 0.155^c^	1.990 ± 0.207^b^	2.350 ± 0.245^a^
35.97	Docosane	m/z 310,211,183,127,99,71,57	ND^c^	4.160 ± 0.433^a^	ND^c^	2.540 ± 0.264^b^
36.35	Linolenic acid	m/z 350,336,321,260,117,108,75,73	2.660 ± 0.277^b^	2.940 ± 0.306^b^	2.500 ± 0.260^b^	14.370 ± 1.496^a^
36.77	Stearic acid	m/z 356,341,146,117,83,73,54	0.980 ± 0.102^c^	1.890 ± 0.197^b^	0.890 ± 0.093^c^	2.700 ± 0.281^a^
37.67	Tricosane	m/z 324,239,169,127,99,85,71,57	ND^c^	2.540 ± 0.264^a^	ND^c^	1.590 ± 0.165^b^
39.31	Tetracosane	m/z 338,309,239,155,127,113,71,57	ND^c^	3.020 ± 0.314^a^	ND^c^	1.850 ± 0.193^b^
40.89	Pentacosane	m/z 352,295,197,155,127,85,71,57	ND^c^	1.740 ± 0.181^a^	ND^c^	1.150 ± 0.120^b^
42.41	Hexacosane	m/z 366,253,155,99,85,71,57	ND^c^	1.650 ± 0.172^a^	ND^c^	0.930 ± 0.097^b^
43.04	Docosanoic acid	m/z 412,398,243,149,129,117,73,55	ND^b^	0.210 ± 0.022^a^	ND^b^	0.230 ± 0.024^a^
43.87	Heptacosane	m/z 380,323,281,127,99,85,71,57	0.620 ± 0.065^b^	1.170 ± 0.122^a^	0.350 ± 0.036^c^	0.690 ± 0.072^b^
44.91	2-Monolinolein	m/z 498,482,408,234,191,129,73,55	2.010 ± 0.209^a^	ND^c^	0.750 ± 0.078^b^	ND^c^
45.29	Octacosane	m/z 394,337,183,127,113,99,85,71,57	ND^c^	1.060 ± 0.110^a^	ND^c^	0.680 ± 0.071^b^
45.86	Tetracosanoic acid	m/z 440,426,381,257,149,129,117,75,73	0.270 ± 0.028^b^	0.180 ± 0.019^c^	0.310 ± 0.032^b^	0.460 ± 0.048^a^
50.02	α-Tocopherol	m/z 502,487,354,237,123,95,73	ND^b^	0.510 ± 0.053^a^	ND^b^	ND^b^
50.17	Cholesterol	m/z 458,416,329,247,129,95,73,57	1.320 ± 0.137^a^	0.820 ± 0.085^b^	0.880 ± 0.092^b^	0.550 ± 0.057^c^
51.51	Campesterol	m/z 472,457,343,289,247,145,73,55	4.750 ± 0.494^b^	1.410 ± 0.147^c^	11.200 ± 1.166^a^	2.150 ± 0.224^c^
51.93	Stigmasterol	m/z 484,355,213,161,129,83,69,73,53	0.940 ± 0.098^c^	1.290 ± 0.134^c^	11.480 ± 1.195^a^	3.890 ± 0.405^b^
52.76	β-Sitosterol	m/z 486,398,275,213,159,129,73,57	34.070 ± 3.546^a^	9.230 ± 0.961^c^	24.850 ± 2.586^b^	11.550 ± 1.202^c^

*Mean values ± SD of mg/g of the dry weight of samples. ND, not detected; a, b, c, and d denote statistical significance P < 0.05. Cont, control; SNP, biogenic silver nanoparticles alone; AB, A. brassicicola-infected plants; AB + SNP, A. brassicicola-infected, treated with SNP.*

**TABLE 2:** Quantitative variability in polar metabolites of *A. thaliana* leaves during plant pathogen nanoparticles interaction.

**Rt (Min)**	**Metabolites**	**Fragmentation patterns**	**Control (μg/g)**	**SNP (μg/g)**	**AB (μg/g)**	**SNP + AB (μg/g)**
6.79	Ethylene glycol	m/z 206,193,191,147,103,73	0.710 ± 0.074^b^	0.270 ± 0.028^d^	0.500 ± 0.052^c^	1.370 ± 0.143^a^
16.07	Glycerol	m/z 308,293,219,205,148,117,75,73,55	1.360 ± 0.142^d^	2.590 ± 0.270^c^	5.990 ± 0.623^b^	9.470 ± 0.986 ^a^
16.72	Maleic acid	m/z 260,247,245,147,75,73,66	ND^b^	0.290 ± 0.030^a^	ND^b^	ND^b^
16.95	Succinic acid	m/z 262,249,247,172,148,129,75,73,55	1.460 ± 0.152^a^	1.300 ± 0.135^a^	ND^b^	ND^b^
17.63	Glyceric acid	m/z 332,308,292,205,189,147,103,73	0.900 ± 0.094^b^	2.140 ± 0.223^a^	ND^c^	ND^c^
17.89	Fumaric acid	m/z 260,248,217,147,133,75,73	ND^b^	11.350 ± 1.18^a^	ND^b^	ND^b^
21.77	Malic acid	m/z 350,335,307,245,148,133,73,55	ND^b^	7.680 ± 0.799^a^	ND^b^	ND^b^
22.39	Erythritol	m/z 410,348,307,217,205,189,147,117,103,73	0.570 ± 0.059^a^	0.251 ± 0.026^c^	ND^c^	0.240 ± 0.02^b^
23.26	Erythronic acid	m/z 424,409,379,294,220,149,117,73	0.280 ± 0.029^a^	0.320 ± 0.03^a^	ND^b^	ND^b^
23.68	L-Threonic acid	m/z 424,409,379,294,220,189,147,117,73	1.060 ± 0.110^b^	3.120 ± 0.325^a^	ND^c^	ND^c^
26.12	D-Xylose	m/z 467,421,362,307,147,103,73,59	1.520 ± 0.158^a^	0.940 ± 0.098^c^	1.220 ± 0.127^b^	0.970 ± 0.10^c^
26.48	D-ribose	m/z 467,467,362,218,189,103,73	4.350 ± 0.453^a^	0.410 ± 0.043^bc^	0.660 ± 0.069^b^	ND^c^
27.38	Xylitol	m/z 512,395,307,217,147,103,73	1.030 ± 0.107 ^b^	0.300 ± 0.031^c^	3.050 ± 0.317^a^	1.240 ± 0.129^b^
28.36	Ribonic acid	m/z 526,511,421,333,292,217,147,103,73	1.680 ± 0.175^b^	2.220 ± 0.231^a^	ND^c^	ND^c^
28.56	Galactonic acid	m/z 628,511,393,292,217,189,147,103,73	ND^b^	1.860 ± 0.194^a^	ND^b^	ND^b^
30.65	D-Fructose (MeOX1)	m/z 569,408,307,263,217,173,147,103,73	2.100 ± 0.219^c^	8.160 ± 0.849^b^	16.840 ± 1.753^a^	ND^d^
30.86	D-Fructose (MeOX2)	m/z 569,408,307,263,217,173,147,103,73	ND^c^	5.840 ± 0.608^b^	11.220 ± 1.168^a^	ND^c^
31.13	D-Glucose (MeOX2)	m/z 569,466,390,319,217,160,147,73	2.480 ± 0.258^c^	14.130 ± 1.47^b^	17.170 ± 1.787^a^	1.160 ± 0.121^c^
31.49	D-Galactose	m/z 569,376,217,160,147,103,73	ND^b^	4.200 ± 0.437 ^a^	4.680 ± 0.487 ^a^	ND^b^
31.76	D-Glucitol	m/z 614,422,346,320,217,157,103,73	ND^c^	0.430 ± 0.045 ^c^	12.370 ± 1.288^a^	4.290 ± 0.447^b^
32.03	Ribitol	m/z 512,379,218,157,103,73,59	ND^b^	ND^b^	1.810 ± 0.188^a^	ND^b^
32.18	Gulonic acid	m/z 466,361,218,147,129,75,73	1.710 ± 0.178^b^	0.150 ± 0.016^c^	2.400 ± 0.250^a^	1.080 ± 0.112^b^
33.16	Gluconic acid (Di TMS) (6TMS)	m/z 628,437,334,292,277,147,73	ND^b^	5.220 ± 0.543 ^a^	0.420 ± 0.044^b^	ND^b^
34.71	Inositol	m/z 612,508,362,337,217,191,147,73	11.550 ± 1.202^b^	3.160 ± 0.329^c^	1.460 ± 0.360^d^	29.910 ± 3.113^a^
38.75	α-D Galactopyranoside (6TMS)	m/z 686,362,217,204,191,147,73	2.320 ± 0.241^b^	2.480 ± 0.258^b^	3.200 ± 0.333 ^b^	18.350 ± 1.910^a^

*Mean values ± SD of μg/g of the dry weight of samples. ND, not detected; a, b, c, and d denote statistical significance P < 0.05. Cont, control; SNP, biogenic silver nanoparticles alone; AB, A. brassicicola-infected plants; AB + SNP, A. brassicicola-infected, treated with SNP.*

The concentration of sterols including β-sitosterol, stigmasterol, and compesterol increased significantly in AB alone as compared to control. Sterols are known to accumulate during pathogen infection, thereby promoting plant disease susceptibility (Berger et al., 2004; Griebel and Zeier, 2010). A significant decrease in β-sitosterol, stigmasterol, and compesterol in AB + SNP as compared to AB treated leaves was observed. Similarly, concentrations of sugars, D-xylose, D-ribose, D-fructose, D-glucose, D-galactose, and sugar alcohols such as ribitol, glucitol, and xylitol were found to increase significantly in infection of AB. Sugar accumulation in plants is a sign of plant’s strategy to fight infection, which can be manipulated and utilized by fungal pathogen to acquire food and nutrition for their survival (Moghaddam and Ende, 2012). Decrease in sugar level in SNP-treated plants is also an evidence of the successful inhibition of plant pathogen by these tiny particles (Table 1).

Hydrocarbons including cyclotetradecane, tetradecane, pentadecane, hexadecane, heptadecane, and non-adecane are known for their antimicrobial activity and have been extracted from many medicinal plants (Ozdemir et al., 2004; Lin et al., 2012). Similarly, eicosane; heneicosane; tri-, tetra-, penta-, hexa-, hepta-, octa-, and docosane; and tetracosanoic acid compounds demonstrate antagonistic activities toward pathogens (Kalegari et al., 2012; Zhang et al., 2014). The levels of these compounds were significantly enhanced in SNP-treated plants. The enhanced concentration of the compounds in SNP treatment demonstrates their positive effect on plant immune response, which may be an important factor for plant survival after fungal infection. The AB + SNP treatment showed increased concentration of these metabolites, indicating preparation of plants to combat biotic stress.

Organic acids of antimicrobial potential (Kabara et al., 1972; Schnürer and Magnusson, 2005) including propanoic acid, caproic acid, lauric acid, docasanoic acid, erythronic acid, L-threonic acid, non-anoic acid, galactonic acid, gluconic acid, and ribonic acid were not detected in control and pathogen-infected plants while silver nanoparticle-treated plants emerged as significant producers of these organic compounds. Similarly, antimicrobial stearic acid (Da Silva et al., 2002) was significantly enhanced with treatment of silver nanoparticles. Compounds having antimicrobial properties not only helped plants to combat the attack of *A. brassicicola* but also acted as vaccines to plants, which keeps them ready to fight any further biotic stress and activated defense mechanism.

TCA cycle intermediates such as maleic acid, succinic acid, glyceric acid, fumaric acid, and malic acid concentration increased in leaves of SNP treatment. The concentrations of pristane and phytane, saturated terpenoids that are known for their antifungal potential (Yin et al., 2011), were also enhanced in leaves treated with silver nanoparticles, whereas they were completely absent in control and pathogen-infected leaves.

Metabolites showing antioxidant potential as phytol and α-tocopherol (Kontush et al., 1996; Santos et al., 2013) were significantly increased in SNP-treated leaves. The concentration of α-D galactopyranoside and inositol was much higher in AB + SNP as compared to other treatments. Inositol acts as an osmolyte, which balances the osmotic pressure during infection (Khan et al., 2012).

### Inductively Coupled Plasma-Mass Spectroscopy (ICP-MS) Analysis

**FIGURE 7:**
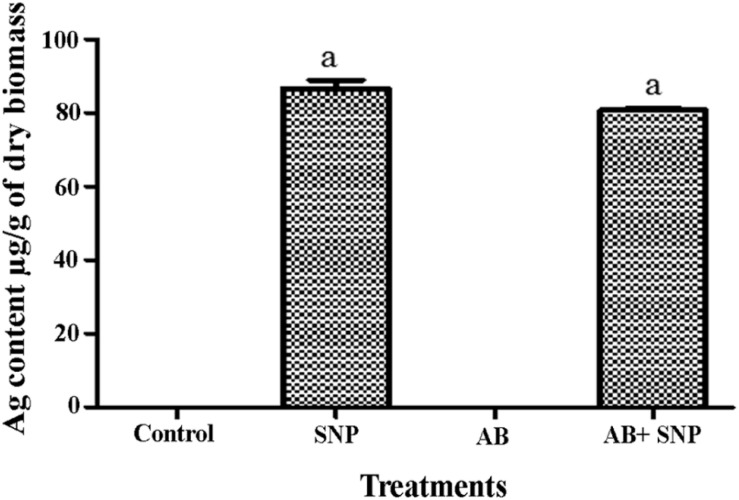
Total Ag content in leaves of *A. thaliana.* Cont, control; SNP, biogenic silver nanoparticles alone; AB, *A. brassicicola*-infected plants; AB + SNP, *A. brassicicola*-infected, treated with SNP. Values are the means ± SD of three replicates. Means sharing different letters “a” and “b” differ significantly from each other at *p* ≤ 0.05.

To study the internalization of silver in leaf tissues, ICP-MS studies were carried out. In both SNP and AB + SNP treatments, the concentration of silver inside the leaves was 87.6 and 80.7 μg/g of dry biomass, respectively (Figure 7). Larue et al. (2014) have also found an accumulation of silver in the leaves of *Lactuca sativa* after foliar exposure without observing any phytotoxicity. Our further studies on the interaction of studied plant pathosystem–silver nanoparticles with soil show that negligible effects were found on native soil microflora (Kumari et al., 2017c). Das et al. (2016) have found iron oxide nanomaterials as a sustainable and eco-friendly source of slow iron release without showing phytotoxicity and effect on seed germination. Agglomeration and oxidation of nanoparticles inside plant cells and in soil might be some of the reasons that nullify the toxic effects of silver nanoparticles on plants. Silver nanoparticle phytotoxicity has been observed by several studies on higher concentration, exclusively after root exposure (Larue et al., 2014); this study using 5 μg/ml SNP by foliar exposure can be considered as safe, though further studies are required to understand the fate of nanoparticles inside cells.

### Mechanism Involved in Silver Nanoparticle-Mediated Plant Protection Against Pathogens

**SCHEME 1:**
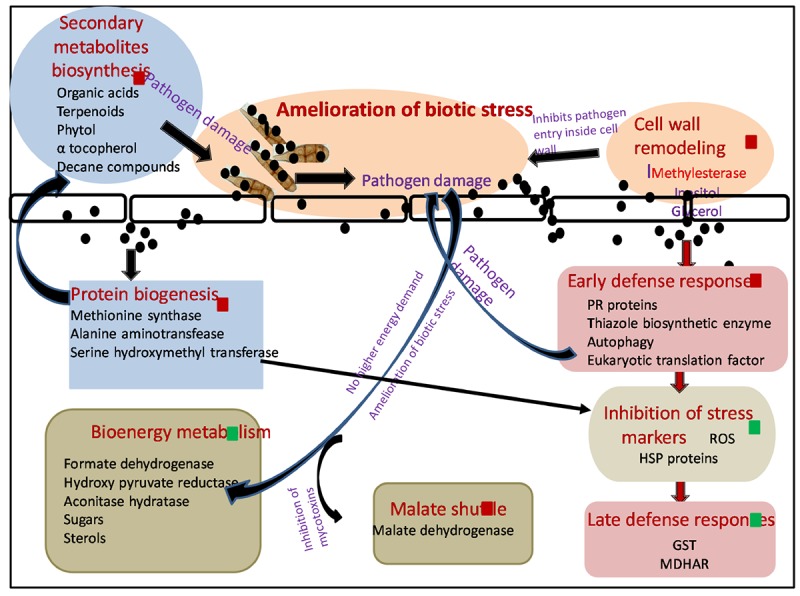
Molecular mechanism involved in amelioration of biotic stress by SNP during tripartite interaction of plant–pathogen–nanoparticles. Black spheres represent SNP, red squares represent proteins and metabolites upregulated, and green squares represent proteins and metabolites downregulated. Red arrows shows the differentially expressed proteins involved in different pathways. Black arrows demonstrate hypothesized mechanism linking differentially expressed proteome and metabolome. ROS, reactive oxygen species; HSP, heat shock proteins; GST, glutathione S transferase; MDHAR, monodehydroascorbatereductase.

Biosynthesized silver nanoparticles not only were able to kill plant pathogen *A. brassicicola* after their foliar spray but also enhanced plants’ immunity employing a variety of mechanisms (Scheme 1).

In this study, *A. brassicicola* infection (AB only) caused upregulation of several proteins involved in BEM and thus increase in sugar and sterol content was observed in GC-MS. Successful establishment of necrotrophic infection starts with damaging plant cells, thereby increasing the energy constraint over the metabolic pathways of the host, which is further utilized by the fungus itself for its nourishment and growth, leading toward the death of host plant (Divon and Fluhr, 2007). Application of SNP resulted in amelioration of biotic stress by damaging fungal pathogen and thus resulting in a downregulation in the expression of proteins involved in sugar metabolism and sugar metabolites as compared to AB-only plants. SNPs were also able to protect the host plant by the mycotoxins released by *A. brassicicola* affecting malate shuttle as observed by an upregulation in the expression of protein malate dehydrogenase after treatment of SNP in comparison with AB alone.

Interaction of SNP with host plant resulted in the elevation of several proteins involved in early defense response of the plants, thus minimizing the production of ROS and collateral damage induced by them. Induction of disease resistance protein, thiazole biosynthetic enzyme, and serine hydroxymethyl transferase resulted in decreased production of ROS and several stress markers such as proline, TBARS, and dnaK-type molecular chaperone hsc70.1 as observed by biochemical and proteomics study. As the production of ROS was minimized by the application of SNP, expression of ROS scavenging enzymes such as GST was also not elevated, while in AB alone, induced biotic stress multifold expression of many GST proteins was observed.

Autophagy also emerged as a probable mechanism that could contribute toward enhanced early defense response of plants against the pathogen after application of SNP. Autophagy plays a very important role in inducing cell death including necrotrophic fungi, depriving them of energy. It also regulates salicylic acid and jasmonic acid, thus influencing plant basal resistance against necrotrophic pathogens (Zhou et al., 2012). Increased level of U-box domain-containing protein 38 and E3 ubiquitin ligase was observed in AB + SNP, indicating the upregulation of proteins involved in autophagy. This result was strongly supported by the TEM images where autophagy and chlorophagy were observed in AB + SNP treatment.

Significant enhancements in proteins involved in protein biogenesis were observed in SNP and SNP + AB treatments. Synthesis of a diverse pool of amino acids and related proteins, especially methionine, can be further utilized in synthesis of antimicrobial proteins, secondary metabolite, and organic acids, which was evident from the results of GC-MS. Higher expression of the protein involved in the amino acid synthesis and methionine biosynthetic pathway in SNP and AB + SNP was able to produce a range of antimicrobial compounds that can fight the pathogen before establishment of infection, thus providing a higher level of immunity to plants. The higher expression level of methyl esterase was also observed in AB + SNP as compared to AB, which might be responsible for enhanced resistance of plant cell wall by maintaining its osmoregulation by increasing the production of osmolytes such as inositol.

## Conclusion

In this study, antifungal activity of biosynthesized silver nanomaterial was tested in the model pathosystem of *A. thaliana* and *A. brassicicola.* SNP demonstrated excellent antimicrobial activity and helped host plant to combat the disease as evident by disease parameters, enzymatic and non-enzymatic assays, and light and electron microscopy. It enhanced the phenolic content; ameliorated biotic stress as observed by a decrease in H_2_O_2_, LPX, and proline content; and subsequently decreased the concerned defense enzyme without showing any phytotoxicity.

Proteomics and metabolomics study of *A. thaliana* leaves during tripartite interaction of plant, pathogen, and nanoparticles revealed that many proteins and metabolites differentially expressed during the interaction. Proteins related to bioenergy, metabolism, and plant defense were most abundant to be differentially expressed, resulting in differential regulation of sugar, steroid, and secondary metabolite synthesis during tripartite interaction. This study provides an insight into the role of SNP in plant disease management. The nanomaterials were not only capable of killing the pathogen; they also enhanced antimicrobial property of plant and induced primary and secondary immune responses by modulating proteome and metabolome.

## Supporting Information

This contains further information regarding characterization of SNP, detached leaf assay, lactophenol staining, stress and antioxidative parameters, 2-DE gel analysis, zoomed-in gel images, reproducibility of 2-DE gels, MS/MS details of identified differential proteins, and putative domain analysis of identified unknown proteins, as well as detailed result and discussion of differentially expressed proteins and detailed methodology of MS/MS and GC-MS studies.

## Data Availability Statement

All datasets generated for this study are included in the article/Supplementary Material.

## Author Contributions

The idea was conceived by CN and AM. The work was done by MK, SP, SM, and VG. The data were interpreted by MK, SP, LA, and AP. The manuscript was written by MK, SP, and VG. The manuscript was critically analyzed by LA, SD, AM, and CN.

## Conflict of Interest

The authors declare that the research was conducted in the absence of any commercial or financial relationships that could be construed as a potential conflict of interest.

## Footnotes

1 http://www.matrixscience.Com

2 http://pfam.sanger.ac.uk

3 http://www.ebi.ac.uk/interpro
