# Supplementary material for: Omics-Based Mechanistic Insight Into the Role of Bioengineered Nanoparticles for Biotic Stress Amelioration by Modulating Plant Metabolic Pathways
Source: Front Bioeng Biotechnol. 2020 Apr 17;8:242. doi: 10.3389/fbioe.2020.00242 (PMC7180193; doi:10.3389/fbioe.2020.00242)
Supplement: Supplementary file 1 [file Data_Sheet_1.pdf]

Supporting Information-

Omics based mechanistic insight into the role of bioengineered nanoparticles for biotic stress amelioration by modulating plant metabolic pathways

Madhuree Kumari[1,3#], Shipra Pandey[1,3#], Shashank Kumar Mishra[1,3],Ved Prakash Giri[1,4], Lalit Agarwal[1,5], Sanjay Dwivedi[1], Alok Kumar Pandey[2], Chandra Shekhar Nautiyal[1], Aradhana Mishra[1*] [1]CSIR-National Botanical Research Institute, Rana Pratap Marg, Lucknow, 226 001, India

[2]CSIR-Indian Institute of Toxicology Research, VishvigyanBhawan 31, Mahatma Gandhi Marg, Lucknow, 226 001, India

[3]Academy of Scientific and Innovative Research (AcSIR), Ghaziabad- 201002, India

[4]Department of Botany, Lucknow University, Hasanganj, Lucknow 226007, India

[5]Department of Agriculture and Allied Sciences, Doon Business School, Dehradun 248011, India

*Corresponding author:

***Dr.Aradhana Mishra**,*
Principal Scientist,
Division of Plant Microbe Interactions,
CSIR- NBRI
Rana Pratap Marg, Lucknow, 226 001, India.
E mail: mishramyco@yahoo.com
Tel: 91 522 2297987, Fax: +91 522 2205839

[#]*These authors contributed equally to this work.*

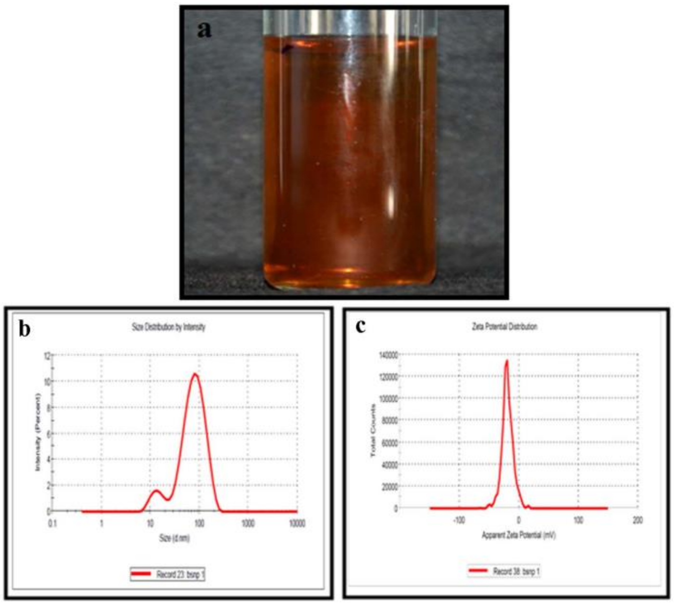

Figure S1. Characterization of biosynthesized silver nanoparticles **(a)** Colour change after formation of silver nanoparticles **(b)** zeta size **(c)** zeta potential

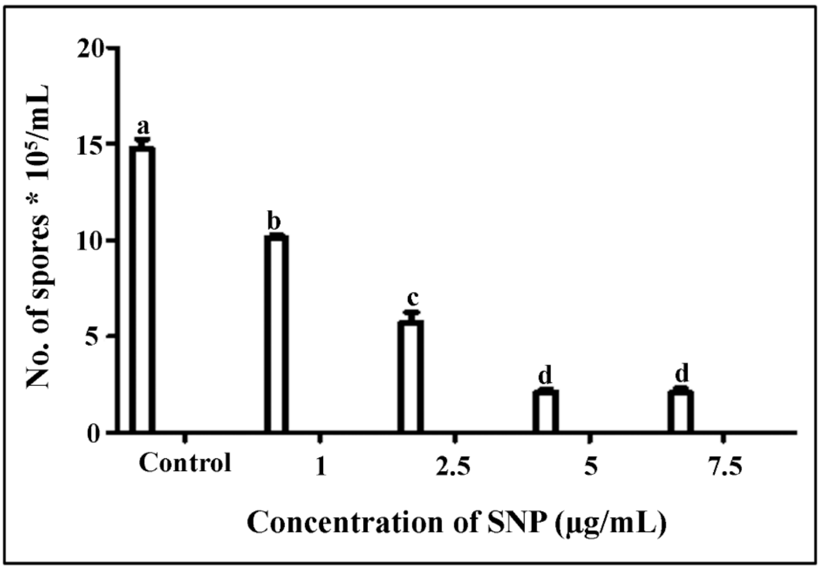

Fig.S2. Detached leaf assay with different concentrations of biogenic silver nanoparticles (AB+SNP) in comparison to AB only control. Values are the means± SD of three replicates. Means sharing different alphabets ‘‘a’’, ‘‘b’’ differ significantly from each other at $p \leq 0.05$.

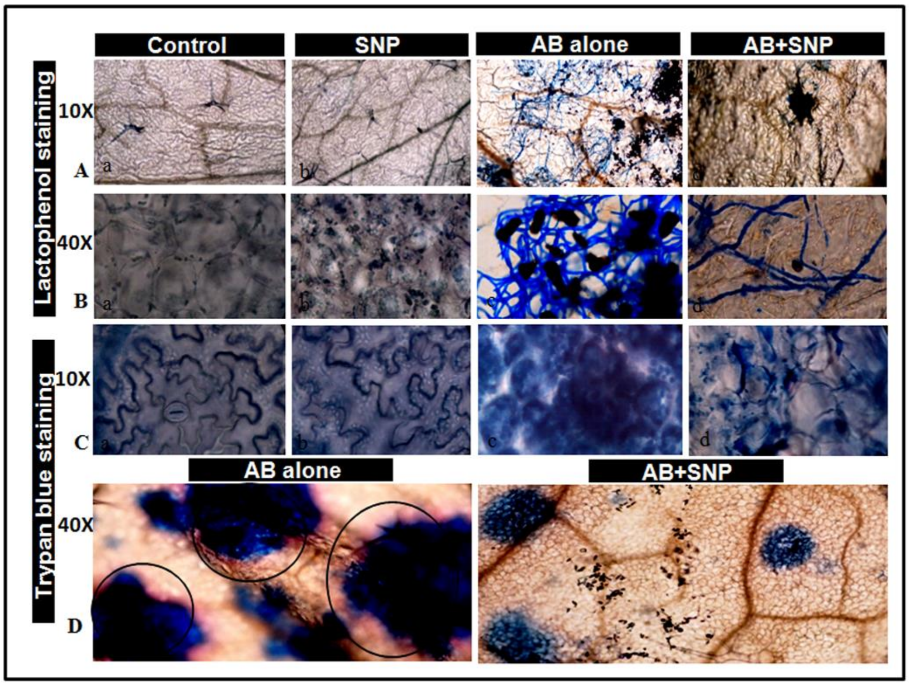

Fig. S3. Micrographs of *A. thaliana* leaves stained with **(A):** lactophenol stained leaves for observation of fungal growth at 10 X in **(a)** control **(b)** SNP **(c)** AB alone **(d)** AB+ SNP**(B):**lactophenol stained leaves for observation of fungal growth at 40 X in **(a)**control **(b)** SNP **(c)** AB alone showing clear mycelial network **(d)** AB+ SNP showing significant reduction in fungal mycelia**(C):** Trypan blue stained leaves for observation of dead parts at 10 X in **(a)** control **(b)** SNP **(c)** AB alone **(d)** AB+ SNP**(D):**Trypan blue stained leaves for observation of dead parts at 40 X in **(a)** AB alone. Encircled area represents dead tissue of leaf due to pathogen invasion **(b)** AB+SNP. Reduction in damage in leaves pre-treated with SNP

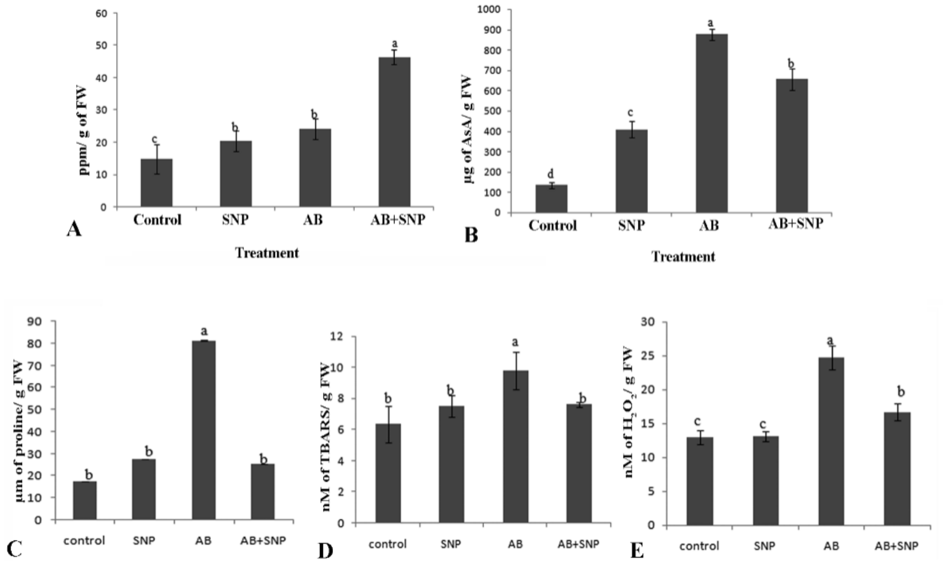

Fig.S4. Antioxidant and stress activities across various treatments in terms of (A) total phenolics (B) ascorbic acid content**(C)**proline **(D)**TBARS content **(E)** H_2O_2 content.Cont-Control, SNP-Biogenic silver nanoparticles lone, AB-*A. brassicicola* infected plants, AB+SNP-*A. brassicicola* infected, treated with SNP. Values are the means± SD of three replicates. Means sharing different alphabets ''a'', ''b'' differ significantly from each other at $p \leq 0.05$.

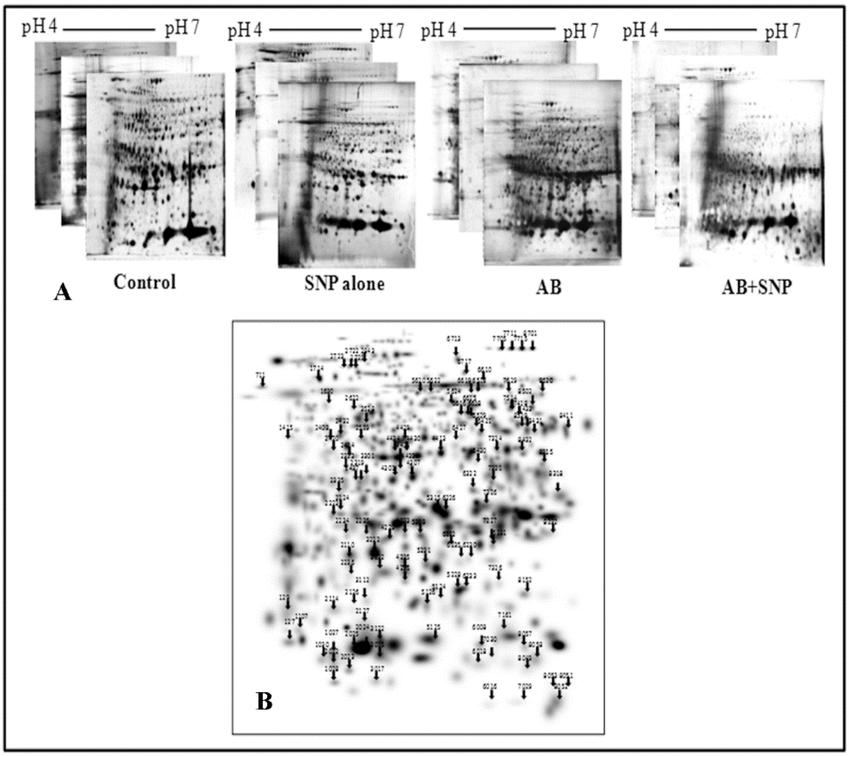

Fig. S5. 2-DE analysis of *Arabidopsis* proteome **(A)** extracted protein by 2-DE and stained by silver gels of different treatments in triplicate **(B)** a higher level match set created from three replicate gels of all treatments using PD Quest analysis by combining them computationally. Arrow indicates differentially expressed proteins across all treatments.Cont-Control, SNP-Biogenic silver nanoparticles lone, AB-*A. brassicicola* infected plants, AB+SNP-*A. brassicicola* infected, treated with SNP.

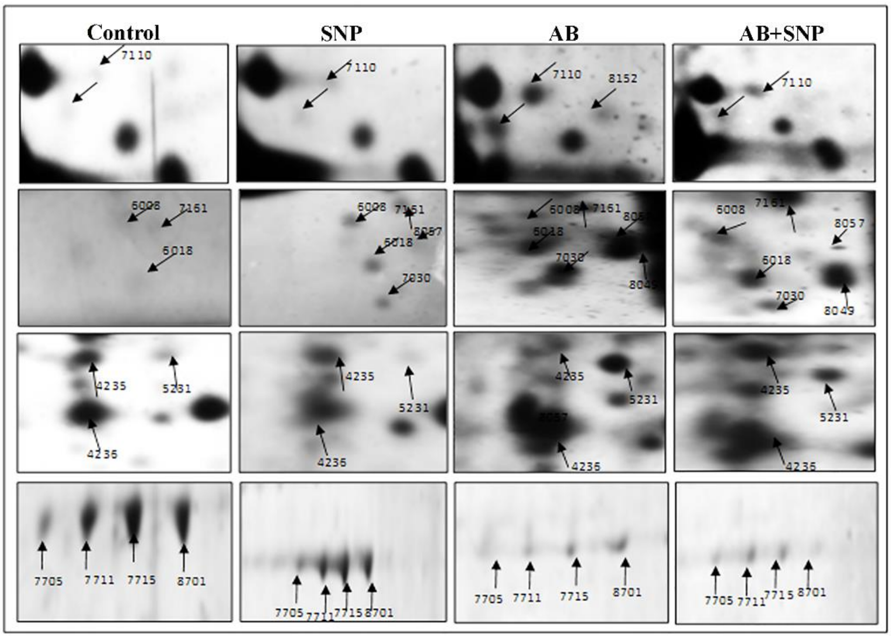

Fig. S6. Zoomed in gel sections of differentially expressed proteins during plant-pathogen nanoparticles interaction. Arrow indicates protein number assigned during PD Quest analysis.Cont-Control, SNP-Biogenic silver nanoparticles lone, AB-*A. brassicicola* infected plants, AB+SNP-*A. brassicicola* infected, treated with SNP.

Materials and Methods

Gel Electrophoresis

Overnight rehydration was carried out with immobilized pH gradients (IPG) strips (13 cm, pH 4-7, linear) with 250 µl of rehydration buffer (8M urea, 2M Thiourea,4% CHAPS, 20 mM DTT, 0.5% biolytes) at room temperature. Isoelectric focusing (IEF) was conducted at 20 °C using an Ettan IPGphore-3 (GE Healthcare) with the conditions provided for rehydration as, 500 V for 60 min, 1000 V for 30 min, 3500 V for 60 min, and 5500 V for 60 min and 6500 V for 4 h for a total of 22000 Total Volt hr. The focused strips were equilibrated twice for 15 min in 10 mL of equilibration solution containing 6 M urea, 30% w/v glycerol, 2% w/v sodium dodecyl sulfate (SDS) and 50 mMTrisHCl buffer, pH 8.8 with 1% w/v DTT and 2.5% w/v iodoacetamide respectively. For the second dimension SDS PAGE, the equilibrated strips were positioned on the 12.5% resolution gel and sealed with 0.5% agarose solution. Gels were stained with silver stain and gel images were acquired with the Chemidoc MP imager (Biorad, USA) with 16 bit CCD camera. The data was analysed using PD quest analysis (PD quest basic 8.0.1, Biorad, USA)

The parameters like protein spot quality, molecular mass, and pI of individual protein was assessed as described earlier by Agrawal et al. (2016). The spots showing reproducibility in quality and quantity in at least two of the three replicate gels were taken in consideration during analysis. A normalized spot volume for protein quantification was obtained by PDQuest software using the total spot volume normalization procedure to avoid experimental variations in gels due to protein load or staining.

Protein identification

Tryptic digestion of the differential protein spots excised from the gels, and sample preparation were performed following the protocols of Agarwal et al.(2016) with some modifications. Briefly, excised spots were washed three times with sterile MQ water to remove impurities and macerated into pieces. Further, gel pieces were destained by 15mM potassium ferricyanideand

50 mM sodium thiosulphate until the colour disappeared the gel pieces dehydrated by acetonitrile and 50mM ammonium bicarbonate in 2:1 ratio. Again supernatant was removed and 25mM of ammonium bicarbonate was added. This process was repeated three times for proper washing. Destained particles were vacuum dried and rehydrated in equal volumes of 0.1 mg/ mL trypsin and 50mM ammonium bicarbonate. Gel particles were immersed in 25 mM ammonium bicarbonate and samples were digested overnight at 37 °C. Peptides were extracted twice with 50% acetonitrile /1% trifluoroacetic acid. The recovered peptides were concentrated to a final volume of 10 µL. The extracted peptides were desalted and concentrated for MALDI analyses using C 18 Zip Tips (Millipore, Schwalbach, Germany).

GC/MS

Thirty m 0.25 mm Thermo TR50 column (polysiloxane column coated with 50% methyl and 50% phenyl groups) was used for chromatographic separation of metabolites. To prepare the sample for GC–MS analysis of polar and non-polar extracts, TMS derivative of the sample was prepared. Approximately 5 mg of the sample was suspended in 40 µl of the methoxylamine hydrochloride in pyridine (20 mg/mL). The mixture was incubated for 4 h at 37 °C in shaking condition before adding 70 µL of the 2,2,2-trifluoro-N-methyl-N-trimethylsilylacetamide (MSTFA). Further, 40 µL of the derivatized solution was subjected to analysis on GC. With an initial 5-min solvent delay time at 70 °C, the oven temperature was increased to 330° C at 5 C/ min, 5 min isocratic and cooled down to 70 °C followed by an additional 5-min delay. Helium flow was maintained at 1 mL/min and split ratio was maintained 1/60. The GC–MS analysis was carried out using Xcalibur version 1.4 software (Thermo Fischer Scientific USA). The GC–MS profile thus obtained was characterized using REPLIB, WILLY and NIST mass spectral library and by matching the chromatogram with standards. The percent peak area of aqueous metabolites was calculated the basis of total numbers of peaks detected in the GC

chromatogram. However, the non-polar metabolites were quantified by plotting regression curve using reference compounds viz. palmitic acid, stearic acid, β-sitosterol and stigmasterol.

Results and Discussion

Characterization of Nanoparticles

The biogenic silver nanoparticles used in this study were spherical (2-5nm) (Kumari et al., 2017b, 2019) and were the most potential antimicrobial agent among different shape and sizes of nanoparticles biosynthesized by *T. viride* cell-free extract as shown in our earlier studies (Kumari et al., 2017b) (Fig. S1a). Further, the zeta size of the particles was observed to be 56.59±0.2nm (Fig. S1b) depicting the coating of nanoparticles with biological moieties of *T. viride*, whereas the zeta potential of the particles was -18.9±0.5mV (Fig. S1c) depicting the stability of particles.

Protein related to bioenergy and metabolism (BEM)

For a variety of biotic and abiotic stress, metabolic configuration and adaptation of plants changes involves a network of molecular pathways to cope up the stress and fulfil higher energy demand (Kushalappa and Gunnaiah, 2013; Ghosh and Xu, 2014) . The proteins involved in BEM were found to be 44% of the total differentially expressed identified protein.

Ferredoxin-NADP (+)-oxidoreductase (AtC-1107), formate dehydrogenase (AtC -8232), UTP-glucose-1-phosphate uridylyltransferase (AtC -6615 and 6616), hydroxypyruvate reductase (AtC -7114), and aconitate hydratase (AtC - 3015), were highly upregulated in infected plants for accomplishing the higher requirement of energy (Bouchartet al., 2007; Muloet al., 2011; Alekseeva et al., 2011; Delleroet al., 2016).

These proteins showed less abundance in AB+SNP treatment as compared to AB alone but were upregulated as compared to control. This kind of expression profiles might be the result

of antifungal activity of silver nanoparticles making plant more resistant to biotic stress with reduced necrosis and chlorosis, resulting in lesser requirement of energy for plant machinery. *Alternaria* sp. invades plant machinery by producing three different types of mycotoxins and the second group of toxin represented by ACR (L)-toxin, induces changes in mitochondria and inhibits malate oxidation. Expression of malate dehydrogenase (AtC-6427, AtC- 8432), activity was significantly reduced in infected plants showing the degradation in malate shuttle by *Alternaria* toxin, however the AB+SNP treatment showed a higher level of expression of malate dehydrogenase presenting the effectiveness of nanoparticles to inhibit *A. brassicicola* effectively. Carbonic anhydrase plays a dual role in plant's adaptation to different abiotic stresses and replenishing the CO_2 supply within plant cells (Kaulet al., 2001). The increased level of different carbonic anhydrase (AtC -2403,2519, 3301, 4307, 5315), in AB+SNP treatment consistently as compared to infected plants indicates the potential role of SNP in amelioration of biotic stress during *A. brassicicola* infection.

Protein related to Plant Defence

The second largest category (20%) of differentially expressed protein comprised of proteins involved in plant defence. Several differentially regulated protein spots were identified as Glutathione S-transferases (GSTs), a major biotic and abiotic stress responsive protein, which is encoded by over 45 divergent genes in previous study in *Arabidopsis* (Mukherjee et al., 2010). Representation of GST family members in several protein spots migrating at similar molecular weight suggests post-translational modifications (Marrset al., 1996). They might arise from an array of changes occurring in host plant including xenobiotics, pathogen attack and oxidative stress (Chivasaet al., 2006). Two differential spots were identified as glutathione S-transferase (AtC- 3017 and 5126), were upregulated in infected plants, while no differential expression was observed in SNP and AB+SNP. The other identified glutathione S-transferase (AtC-5125, 5229, 7336 and 8051) were highly upregulated in pathogen infected plants (AB)

as compared to control while AB+SNP demonstrated intermediate expression pattern between control and infected counterpart. Enhanced expression of different GST proteins after pathogen attack on *A. thaliana* signifies the oxidative stress faced by the plant while the decreased expression level after treatment of silver nanoparticles specifies the effective role of particles in minimising the oxidative stress caused by *A. brassicicola.*

Monodehydroascorbate reductase AtC-7514), and putative dnaK-type molecular chaperone hsc70.1 (AtC-1630), also showed enhanced expression profile in fungal infected plants while no changes were observed in silver nanoparticles treated plants. In plants, the monodehydroascorbate reductase (MDAR) acts as an important component of the glutathione-ascorbate cycle after generation of reactive oxygen species, catalyzing the conversion of monodehydroascorbate to ascorbate. Any kind of biotic or abiotic stress induces necessary changes to regulate proper folding, modification and secretion of proteins, many of which are involved in combating pathogen attack (Kaulet al., 2011). Elevated expression of heat shock protein serves as a stress marker (Cazaleet al., 2009).

The expression profiles of glutamate dehydrogenase (AtC-2622), and putative disease resistance protein (AtC-6322), exhibited significant upregulation in SNP and AB+SNP, and found to be downregulated in AB only.

Some disease resistant genes recognize specific pathogen effectors molecules that are produced during the infection process at very initial stage of their attack (Martin et al., 2003) and effectively inhibit pathogen growth with minimal collateral damage to the plant (McDowell et al., 2003).Enhanced expression profile of disease resistance protein in silver nanoparticles treated plants demonstrates the ability of particles to enable plant for identifying the pathogen at very early stage and eliminating them before the infection establishment and that might be resulting in down regulation of proteins involved in late defence responses to combat oxidative stress.

Protein biogenesis

The third largest category of differentially regulated proteins comprised of proteins involved in storage and protein biogenesis. Spots AtC 2437 and 2439 were identified as N-glyceraldehyde-2-phosphotransferase, which was upregulated in SNP, AB and AB+SNP as compared to control. The upregulation of this protein has been reported during biotic and abiotic stress (Jiaet al., 2015) where it may play an important role in ameliorating stress by modulating ethylene biosynthetic pathway. The level of expression of thiazole biosynthetic enzyme (AtC-4301), was lowest in infected leaves (AB), followed by AB+SNP, SNP and control. This protein catalyzes the conversion of NAD and glycine to adenosine diphosphate 5-(2-hydroxyethyl)-4-methylthiazole-2-carboxylic acid (ADT), an adenylated thiazole intermediate, which acts as a precursor of thiamine. Thiamine provides tolerance to plants from stress by decreasing the production of reactive oxygen species and hydrogen peroxide (Tunc-Ozdemiret al., 2009). A significant increase in expression level of serine hydroxymethyltransferase (AtC-7418), was obtained in SNP and AB+SNP treated leaves as compared to control. This enzyme catalyses the reversible reaction to convert L-serine to glycine and tetrahydrofolate to 5,10-methylenetetrahydrofolate. It also plays a vital role in photorespiratorypathway, minimizing the production of reactive oxygen species generated by biotic and abiotic stress (Moreno et al., 2005). Higher expression of these proteins in SNP and AB+SNP enhances plant’s first line of defence by minimising ROS production, thereby limiting the damage caused by pathogen.

Some of proteins related to amino acid biosynthesis like putative methionine synthase and alanine aminotransferase were found to be upregulated in SNP treated plants may be utilized by plant in many ways to synthesize proteins and antimicrobial metabolites to enhanced resistance in *Arabidopsis.*

Methionine synthases are responsible for the regeneration of methionine from homocysteine which is very important constituent of proteins, performs diverse functions in translation and formation of several metabolites. Alanine aminotransferase catalyses the reversible transfer of an amino group from glutamate to pyruvate to form 2-oxoglutarate and alanine, occupies a central position in amino acid biosynthetic pathways and the methionine chain elongation cycle of aliphatic glucosinolate formation. Increased level of enzymes and subsequent synthesis of antimicrobial metabolites prepares the plant to combat the pathogen attack at a very early stage, before a successful invasion over host could be achieved.

Similar pattern of expression was obtained for methylesterase (AtC-2223), however the expression level in AB+SNP was higher as compared to AB alone. It also demonstrates the ability of nanoparticles in boosting the plant defence by methyl esterifying plant cell wall which can withstand the attack of hydrolytic enzymes secreted by fungi (Volpiet al., 2011). Another protein, adenosy lhomocysteinase (AtC-7226) was significantly upregulated in AB+SNP which regulates the intracellular concentration of adenosylhomocysteine regulating methylation pattern of many important cellular substrates (Pawłowski, 2009).

A similar trend, downregulation of proteins in infected plants was obtained in this category. To cope up with stress, plant must downregulate protein synthesis in order to conserve cellular resources (Immanuel et al., 2012). Upregulation of proteins in SNP treatment might be a reason behind the increased synthesis of antimicrobial compounds. Synthesis of antimicrobial compounds need involvement of many biosynthetic pathway requiring participation of many enzymes and proteins which can only be fulfilled by their higher expression.

Cell signalling

Proteins involved in cell signalling represented 10% differentially expressed proteins. An elevated E3 ubiquitin-protein ligase (AtC-2236), was found in AB+SNP treatment. U-box domain-containing protein 38 (Atc-7326 and 2236), which also functions as an E3 ubiquitin

ligase (Yan et al., 2003)was found to be upregulated in SNP and AB+SNP treatments. E3 ubiquitin-protein ligase of different families is positive regulators of plant disease resistance (Yu et al., 2003). Their up regulation by more than 5 fold in AB+SNP demonstrates the positive role of silver nanoparticles in recognition of pathogen at very first stage and recruiting the right machinery to get rid of it prior to establishment of infection. E3 ubiquitin-protein ligase also plays an important role in regulation of autophagy (Kuanget al., 2013)in accordance with the observation of chlorophagy in TEM.

Rop guanine nucleotide exchange factor 1(AtC-7161), which is a major signal regulator in plants performing diverse functions including hormone and stress responses (Guet al., 2004) were up regulated in AB and AB+SNP treatments. Nucleoside diphosphate kinase (AtC- 8152), probable inactive receptor kinase (AtC-1027) and adenosine kinase 1(AtC-2420), were significantly upregulated in AB alone, are involved in several cell signalling processes which got triggered by biotic stress.

Miscellaneous functions

Some of the differentially expressed proteins were also found to be associated with miscellaneous functions. Protein spots AtC- 2024, 3015, 8529 and 8431 represented uncharacterized protein F1O19.10/F1O19.10 while hypothetical proteins were signified by AtC 8411, 1030, 4229, 7415, 2235 and 6230. MD-2-related lipid recognition domain-containing protein was significantly upregulated in AB+SNP while the expression level decreased drastically in leaves infected with *A. brassicicola*. The MD-2-related lipid-recognition (ML) domain (AtC- 0129) is involved in lipid recognition, particularly in the recognition of pathogen related products.

References:

Agarwal, L., Gupta, S., Mishra, S. K., Pandey, G., Kumar, S., Chauhan, P. S., Chakrabarty, D., and Nautiyal, C.S. (2016). Elucidation of complex nature of PEG induced drought-stress response in rice root using comparative proteomics approach. *Front. Plant Sci.* 7, 1466.https://doi.org/10.3389/fpls.2016.01466.

Alekseeva, A. A., Savin, S. S., Tishkov,V. I. (2011) NAD + -dependent formate dehydrogenase from plants. *Acta Naturae*. 3, 38–54.

Bouchart, F., Delangle, A., Lemoine, J., Bohin, J., Lacroix, J. (2007). Proteomic analysis of a non-virulent mutant of the phytopathogenic bacterium *Erwinia chrysanthemi* deficient in osmoregulated periplasmic glucans: change in protein expression is not restricted to the envelope, but affects general metabolism. *Microbiol*. 153, 760-767.

Cazale, A., Cle´ment, M., Chiarenza, S., Roncato, M., Pochon, N., Creff, N., Marin, E., Leonhardt, N, D. Noe¨ L. (2009). Altered expression of cytosolic/nuclear HSC70-1 molecular chaperone affects development and abiotic stress tolerance in *Arabidopsis thaliana*. *J. Exp. Bot*. 60, 2653-2664.

Chivasa, S., Hamilton, J. M., Pringle, R. S., Ndimba, B. K., Simon, W. J., Lindsey, K., Slabas, A. R. (2006). Proteomic analysis of differentially expressed proteins in fungal elicitor-treated *Arabidopsis* cell cultures. *J. Exp. Bot*. 57, 1553-1562.

Dellero, Y., Jossier, M., Schmitz, J., Maurino, V.G., Hodges, M. (2016). Photorespiratory glycolate–glyoxylate metabolism.*J. Exp. Bot*. 67, 3041-3052.

Ghosh, D., Xu, J. (2014. Abiotic stress responses in plant roots: a proteomics perspective. *Front. Plant Sci*.5, 1

Gu, Y., Wang, Z., Yang, Z. (2004). ROP/RAC GTPase: an old new master regulator for plant signalling. *Curr. Opin. Plant Biol*. 7, 527-536.

Immanuel, T. M., Greenwood, D.R., MacDiarmid, R. M. (2012). A critical review of translation initiation factor eIF2α kinases in plants - regulating protein synthesis during stress. *Func. Plant Biol*. 39, 717-735.

Jia, H., Shao, M., He, Y., Guan, R., Chu, P., Jiang, H. (2015). Proteome dynamics and physiological responses to short-term salt stress in *Brassica napus* leaves. PLoS ONE. 10, e0144808.

Kaul, T., Reddy, P. S., Mahanty, S., Thirulogachandar, V., Reddy, R. A., Kumar, B., Sopory, S. K., Redd, M. K. (2011). Biochemical and molecular characterization of stress-induced β-carbonic anhydrase from a C (4) plant, *Pennisetum glaucum*.*J. Plant Physiol*. 168, 601-610.

Kuang, E., Qi, J., Ronai, Z. (2013). Emerging roles of E3 ubiquitin ligases in autophagy. *Trends Biochem. Sci*. 38, 453-460.

Kumari, M., Pandey, S., Giri, V. P., Bhattacharya, A., Shukla, R., Nautiyal, C. S., and Mishra, A. (2017b). Tailoring shape and size of biogenic silver nanoparticles to enhance antimicrobial efficacy against MDR bacteria. Microbial Pathogenesis. 105,346-355.

Kumari, M., Giri, V.P., Pandey, S., Kumar, M., Katiyar, R., Nautiyal, C.S., Mishra, A. (2019). An insight into the mechanism of antifungal activity of biogenic nanoparticles than their chemical counterparts. Pesticide Physiology and Biochemistry. 157,45-52. DOI:10.1016/j.pestbp.2019.03.005

Kushalappa, A. C., Gunnaiah, R. (2013). Metabolo-proteomics to discover plant biotic stress resistance genes. *Trends Plant Sci*. 18, 522-531

Kwona, O. K., Sima, J., Kima, S. J., Oh, H. R.,Nam, D. H., Lee, S. (2016). Global proteomic analysis of protein acetylation affecting metabolic regulation in *Daphnia pulex. Biochimie*. 121, 219–227

Marrs, K. A. (1996). The functions and regulation of glutathione S-transferases in plants.*Annu. Rev. Plant Physiol*. 47, 127-58.

Martin, G. B., Bogdanove, A.J., Sessa, G. (2003). Understanding the functions of plant disease resistance proteins. *Annu. Rev. Plant Biol*. 54, 23-61.

McDowell, J. M., Woffenden, B. J. (2003). Plant disease resistance genes: recent insights and potential applications. *Trends Biotechnol*. 21, 178-183.

Moreno, J. I., Martín, R., Castresana, C. (2005). *Arabidopsis* SHMT1, a serine hydroxymethyltransferase that functions in the photorespiratory pathway influences resistance to biotic and abiotic stress. *Plant J*. 41, 451-63.

Mukherjee, A. K., Carp, M., Zuchman, R., Ziv, T., Horwitz, B. A. (2010). Gepstein, S. Proteomics of the response of *Arabidopsis thaliana* to infection with *Alternaria brassicicola*. *J. Proteomics*. 73, 709-720.

Mulo, P. (2011). Chloroplast-targeted ferredoxin-NADP(+) oxidoreductase (FNR): structure, function and location. *Biochim. Biophys. Acta*. 1807, 927-934.S4

Pawłowski, T. Z. (2009). Proteome analysis of Norway maple (*Acer platanoides* L.) seeds dormancy breaking and germination: influence of abscisic and gibberellic acids. *BMC Plant Biol*.9, 48.

Radomiljac, J. D., Whelan, J., Van, der M. M. (2013). Coordinating metabolite changes with our perception of plant abiotic stress responses: emerging views revealed by integrative—omicanalyses. *Metabolites*. 3, 761-786.

Tunc-Ozdemir, M., Miller, G., Song, L., Kim, J., Sodek, A., Koussevitzky, S., Misra, M.N., Mittler, R., Shintani, D. (2009). Thiamin confers enhanced tolerance to oxidative stress in *Arabidopsis*.*Plant Physiol*. 51, 421-432.

Volpi, C., Janni. M., Lionetti, V., Bellincampi, D., Favaron, F., D'Ovidio, R. (2011). The ectopic expression of a pectin methyl esterase inhibitor increases pectin methyl esterification and limits fungal diseases in wheat. *Mol. Plant Microbe Interact*. 24, 1012-1019.

Yan, J., Wang, J., Li, Q., Hwang, J.R., Patterson, C., Zhang, H. (2003). AtCHIP, a u-box-containing e3 ubiquitin ligase, plays a critical role in temperature stress tolerance in *Arabidopsis*. *Plant Physiol*. 132, 861-869.

Yu, Y., Xu, W., Wang, J., Wang, L., Yao, W., Yang, Y., Xu, Y., Ma, F., Du, Y., Wang, Y. (2003). The Chinese wild grapevine (*Vitis pseudoreticulata*) E3 ubiquitin ligase erysiphenecator-induced RING finger protein 1 (EIRP1) activates plant defense responses by inducing proteolysis of the VpWRKY11 transcription factor. *New Phytol*. 200, 834-846.
