# Supplementary material for: Omics-Based Mechanistic Insight Into the Role of Bioengineered Nanoparticles for Biotic Stress Amelioration by Modulating Plant Metabolic Pathways
Source: Front Bioeng Biotechnol. 2020 Apr 17;8:242. doi: 10.3389/fbioe.2020.00242 (PMC7180193; doi:10.3389/fbioe.2020.00242)
Supplement: Supplementary file 2 [file Table_1.DOCX]

| **Sample** | **Average no of spots^a^** | **High quality spots^b^** | **Reproducibility (%)** |
| --- | --- | --- | --- |
| Control | 406 | 403 | 99.26 |
| SNP  AB alone  AB+SNP | 371  445  382 | 369  434  373 | 99.4  97.52  97.64 |

Table S1 Reproducibility of 2DE gels

a = Average number of spots present in three replicate gels of each time point.

b = Spots having quality score more than 30 assigned by PDQuest (Ver.7.2.0).
