# Supplementary material for: Omics-Based Mechanistic Insight Into the Role of Bioengineered Nanoparticles for Biotic Stress Amelioration by Modulating Plant Metabolic Pathways
Source: Front Bioeng Biotechnol. 2020 Apr 17;8:242. doi: 10.3389/fbioe.2020.00242 (PMC7180193; doi:10.3389/fbioe.2020.00242)
Supplement: Supplementary file 3 [file Table_2.DOCX]

| **Sample ID No.** | **Protein Name** | **Accession No.** | **Score** | **MW/Pi** | **Sequence coverage** | **Peptide no.** | **Protein expression profile** | **Matched sequence** |
| --- | --- | --- | --- | --- | --- | --- | --- | --- |
| AtC 2025 | ribulosebisphosphate carboxylase [*A.thaliana*] | gi\|16195 | 219 | 20586/8.22 | 33 | 5 |  | R.IIGFDNTR.Q  K.EYPGAFIR.I  R.EHGNTPGYYDGR.Y  K.LPLFGCTDSAQVLK.E  R.QVQCISFIAYKPPSFTEA.- |
| AtC 2126 | ribulosebisphosphate carboxylase [*A.thaliana*] | gi\|16194 | 140 | 20588/7.59 | 25 | 4 |  | R.IIGFDNTR.Q  K.EYPGAFIR.I  R.EHGNTPGYYDGR.Y  R.QVQCISFIAYKPPSFTEA.- |
| AtC 2127 | ribulosebisphosphate carboxylase [*A.thaliana*] | gi\|16194 | 151 | 20303/7.59 | 12 | 3 |  | K.EVDYLLR.N  R.IIGFDNTR.Q  K.EYPGAFIR.I |
| AtC 3222 | ribulosebisphosphate carboxylase [*A.thaliana*] | gi\|1944432 | 358 | 48008 /6.12 | 11 | 6 |  | K.DTDILAAFR.V  K.ALAALRLEDLR.I  K.LTYYTPEYETK.D  R.LEDLRIPPAYTK.T  K.TFQGPPHGIQVER.D  K.LTYYTPEYETKDTDILAAFR.V |
| AtC 4430 | ribulosebisphosphate carboxylase [*A.thaliana*] | gi\|1944432 | 50 | 48008/6.12 | 6 | 3 |  | R.AVYECLR.G  K.DTDILAAFR.V  R.DLAVEGNEIIR.- |
| AtC 8052 | ribulosebisphosphate carboxylase [*A.thaliana*] | gi\|16194 | 42 | 20588/7.59 | 10 | 2 |  | K.EVDYLLR.N  R.EHGNTPGYYDGR.Y |
| AtC 711 | ribulose-1,5-bisphosphate carboxylase [*Coprosmapumila*] | gi\|1770182 | 195 | 52555/6.13 | 5 | 4 |  | R.DLAVEGNEIIR.Q   K.TFQGPPHGIQVER.D   K.TCQGPPHGIQVER.D   R.NEGRDLAVEGNEIIR.Q |
| AtC 2721 | ribulose 1,5-bisphosphate carboxylase [*Cacosmiarugosa*] | gi\|289890 | 69 | 53003/6.09 | 4 | 2 |  | K.DTDILAAFR.V  R.DLAVEGNEIIR.E |
| AtC 6016 | ribulose-1,5-bisphosphate carboxylase/oxygenase [*Lomatiasilaifolia*] | gi\|4098550 | 62 | 50272/6.43 | 2 | 2 |  | K.DTDILAAFR.V  K.DIDILAAFR.V |
| AtC 2335 | Full=Ribulosebisphosphate carboxylase large chain; | gi\|131974 | 164 | 52139/6.23 | 9 | 5 |  | R.AVYECLR.G  K.DTDILAAFR.V  R.VALEACVKAR.N  K.DDENVNSQPFMR.W  R.GGLDFTKDDENVNSQPFMR.W |
| AtC 7423 | ribulose-1,5-bisphosphate carboxylase/oxygenase large subunit [*A.thaliana*] | [gi\|7525041](http://www.matrixscience.com/cgi/master_results.pl?file=..%2Fdata%2F20140809%2FFTgAoaume.dat#Hit1) | 376 | 52922/5.88 | 13 | 5 |  | R.DLAVEGNEIIR.E  R.LSGGDHIHAGTVVGK.L  R.NEGRDLAVEGNEIIR.E  K.EITFNFPTIDKLDGQE.-  R.GGLDFTKDDENVNSQPFMR.W |
| AtC 3112 | ribulose-1,5-bisphosphate carboxylase/oxygenase large subunit [*Juncusgerardii*] | [gi\|30421654](http://www.matrixscience.com/cgi/protein_view.pl?file=..%2Fdata%2F20140221%2FFTnplzueE.dat&hit=gi%7C30421654&db_idx=1&px=1&ave_thresh=52&_ignoreionsscorebelow=0&report=5&_sigthreshold=0.05&_msresflags=1025&_msresflags2=2&percolate=-1&percolate_rt=0&_minpeplen=7&sessionID=guest_guestsession) | 204 | 46421/6.42 | 7 | 4 |  | K.DTDILAAFR.V  K.DIDILAAFR.V  K.LTYYTPEYETK.D  K.TFQGPPHGIQVER.D |
| AtC 3232 | ribulose-1,5-bisphosphate carboxylase/oxygenase large subunit [*Juncusgerardii*] | [gi\|30421654](http://www.matrixscience.com/cgi/protein_view.pl?file=..%2Fdata%2F20140129%2FFTnmOrSne.dat&hit=gi%7C30421654&db_idx=1&px=1&ave_thresh=47&_ignoreionsscorebelow=0&report=5&_sigthreshold=0.05&_msresflags=1025&_msresflags2=2&percolate=-1&percolate_rt=0&_minpeplen=7&sessionID=guest_guestsession) | 221 | 46421/6.42 | 7 | 4 |  | K.DTDILAAFR.V  K.DIDILAAFR.V   K.LTYYTPEYETK.D   K.TFQGPPHGIQVER.D |
| AtC 3843 | ribulose-1,5-bisphosphate carboxylase/oxygenase large subunit [*Juncusgerardii*] | gi\|30421654 | 137 | 46421/6.42 | 5 | 3 |  | K.DTDILAAFR.V   K.DIDILAAFR.V   K.TFQGPPHGIQVER.D |
| AtC 7030 | ribulose-1,5-bisphosphate carboxylase/oxygenase large subunit [*Juncusgerardii*] | [gi\|30421654](http://www.matrixscience.com/cgi/protein_view.pl?file=..%2Fdata%2F20140221%2FFTnplzueE.dat&hit=gi%7C30421654&db_idx=1&px=1&ave_thresh=52&_ignoreionsscorebelow=0&report=5&_sigthreshold=0.05&_msresflags=1025&_msresflags2=2&percolate=-1&percolate_rt=0&_minpeplen=7&sessionID=guest_guestsession) | 204 | 46421/6.42 | 7 | 4 |  | K.DTDILAAFR.V  K.DIDILAAFR.V  K.LTYYTPEYETK.D  K.TFQGPPHGIQVER.D |
| AtC 6231 | ribulose-1,5-bisphosphate carboxylase/oxygenase large subunit [*Lepidozialaevifolia*] | [gi\|146229220](http://www.matrixscience.com/cgi/protein_view.pl?file=..%2Fdata%2F20140123%2FFTnmIfHOR.dat&hit=gi%7C146229220&db_idx=1&px=1&ave_thresh=52&_ignoreionsscorebelow=0&report=5&_sigthreshold=0.05&_msresflags=1025&_msresflags2=2&percolate=-1&percolate_rt=0&_minpeplen=7&sessionID=guest_guestsession) | 163 | 46496/6.18 | 7 | 4 |  | K.DTDILAAFR.M  K.KTDILAAFR.M  R.LTYYTPEYETK.X  K.TFQGPPHGIQVER.D |
| AtC 7227 | ribulose-1,5-bisphosphate carboxylase/oxygenase large subunit [*Lepidozialaevifolia*] | [gi\|146229220](http://www.matrixscience.com/cgi/protein_view.pl?file=..%2Fdata%2F20140123%2FFTnmIfHae.dat&hit=gi%7C146229220&db_idx=1&px=1&ave_thresh=52&_ignoreionsscorebelow=0&report=5&_sigthreshold=0.05&_msresflags=1025&_msresflags2=2&percolate=-1&percolate_rt=0&_minpeplen=7&sessionID=guest_guestsession) | 182 | 46496/6.18 | 7 | 4 |  | K.DTDILAAFR.M  K.KTDILAAFR.M  R.LTYYTPEYETK.X  K.TFQGPPHGIQVER.D |
| AtC 2622 | ribulose-1,5-bisphosphate carboxylase/oxygenase large subunit [*Erodiumbrachycarpum*] | [gi\|93132998](http://www.matrixscience.com/cgi/protein_view.pl?file=..%2Fdata%2F20140222%2FFTnplbYEL.dat&hit=gi%7C93132998&db_idx=1&px=1&ave_thresh=52&_ignoreionsscorebelow=0&report=0&_sigthreshold=0.05&_msresflags=1025&_msresflags2=2&percolate=0&percolate_rt=0&_minpeplen=7&sessionID=guest_guestsession) | 56 | 52237/6.46 | 4 | 2 |  | K.DTDILAAFR.V  R.DLATDGNAIIR.K |
| AtC 3235 | ribulose-1,5-bisphosphate carboxylase/oxygenase large subunit [*Kunhardtiaradiata*] | gi\|4104575 | 209 | 52526/6.13 | 7 | 4 |  | K.DTDILAAFR.V  K.LTYYTPEYETK.D  K.TFQGPPHGIQVER.D  K.IFQGPPHGIQVER.D |
| AtC 5622 | ribulose-1,5-bisphosphate carboxylase/oxygenase large subunit [*Pentaslanceolata*] | [gi\|294117](http://www.matrixscience.com/cgi/protein_view.pl?file=..%2Fdata%2F20140424%2FFTnTCGYER.dat&hit=gi%7C294117&db_idx=1&px=1&ave_thresh=52&_ignoreionsscorebelow=0&report=0&_sigthreshold=0.05&_msresflags=1025&_msresflags2=2&percolate=-1&percolate_rt=0&_minpeplen=7&sessionID=guest_guestsession) | 93 | 53478/6.13 | 6 | 3 |  | K.DTDILAAFR.V  R.VALEACVKAR.N  R.DLAVEGNEIIR.E |
| AtC 6610 | ribulose-1,5-bisphosphate carboxylase/oxygenase large subunit [*Koeberliniaspinosa*] | [gi\|7240297](http://www.matrixscience.com/cgi/protein_view.pl?file=..%2Fdata%2F20140424%2FFTnTCGEOh.dat&hit=gi%7C7240297&db_idx=1&px=1&ave_thresh=65&_ignoreionsscorebelow=0&report=0&_sigthreshold=0.05&_msresflags=1025&_msresflags2=2&percolate=-1&percolate_rt=0&_minpeplen=7&sessionID=guest_guestsession) | 414 | 52270/6.08 | 15 | 7 |  | K.LIEGVPETLDMLR.A  R.AVYECLR.G  K.DTDILAAFR.V  R.VAIEACVQAR.N  R.DLAVEGNEIIR.E  R.ESTLGFVDLLR.D  K.LNYYTPEYETK.D |
| AtC 6513 | ribulose-1,5-bisphosphate carboxylase/oxygenase large subunit [*Pentadiplandrabrazzeana*] | [gi\|1054917](http://www.matrixscience.com/cgi/protein_view.pl?file=..%2Fdata%2F20140424%2FFTnTCGYEL.dat&hit=gi%7C1054917&db_idx=1&px=1&ave_thresh=65&_ignoreionsscorebelow=0&report=0&_sigthreshold=0.05&_msresflags=1025&_msresflags2=2&percolate=-1&percolate_rt=0&_minpeplen=7&sessionID=guest_guestsession) | 391 | 53290/6.13 | 18 | 7 | \|  \| \| --- \| | K.DTDILAAFR.V  R.DNGLLLHIHR.A  R.DLAVEGNEIIR.E  R.LSGGDHIHAGTVVGK.L  K.TFQGPPHGIQVER.D  R.GGLDFTKDDENVNSQPFMR.W |
| AtC 8058 | ribulose-1,5-bisphosphate carboxylase/oxygenase large subunit [*Brassica rapa* subsp. pekinensis] | [gi\|21311588](http://www.matrixscience.com/cgi/protein_view.pl?file=..%2Fdata%2F20140129%2FFTnmOauSR.dat&hit=gi%7C21311588&db_idx=1&px=1&ave_thresh=46&_ignoreionsscorebelow=0&report=5&_sigthreshold=0.05&_msresflags=1025&_msresflags2=2&percolate=-1&percolate_rt=0&_minpeplen=7&sessionID=guest_guestsession) | 217 | 16104/7.66 | 30 | 3 |  | R.LEDLRIPPAYTK.T  K.TFQGPPHGIQVER.D  SSTGTWTTVWTDGLTSLDR.YA |
| AtC 2323 | Sedoheptulose-1,7-bisphosphatase [*A.thaliana*] | gi\|15228194 | 80 | 42388/6.17 | 10 | 4 |  | K.MFSPGNLR.A  R.ATFDNSEYSK.L  R.FEETLYGTSR.L  K.GIFTNVTSPTAK.A |
| AtC 2432 | Sedoheptulose-1,7-bisphosphatase [*A.thaliana*] | gi\|15228194 | 212 | 42787/6.17 | 15 | 6 |  | R.TLLMCMGEALR.T  R.ATFDNSEYSK.L  R.YTGGMVPDVNQIIVK.E  R.YTGGMVPDVNQIIVK.E + Oxidation (M)  K.TIINLDDRTQVAYGSK.N  R.FEETLYGTSR.L |
| AtC 4233 | fructose-bisphosphatealdolase [*A.thaliana*] | gi\|15231715 | 179 | 38858/6.05 | 31 | 7 |  | K.FADELIANAAYIGTPGK.G  K.GILAADESTGTIGKR.L  R.LASINVENVETNRR.N  K.GTVELAGTDGETTTQGLDGLGDR.C  K.VSPEVIAEHTVR.A  R.TVPAAVPAIVFLSGGQSEEEATR.N  K.AAQEALYVR.C |
| AtC 4419 | putative fructose bisphosphatealdolase [*A.thaliana*] | gi\|14334740 | 261 | 43033/6.48 | 13 | 6 |  | K.RLASIGLENTEANR.Q  R.LASIGLENTEANR.Q  R.TAAYYQQGAR.F  K.EAAWGLAR.Y  R.ATPEQVASYTLK.L  K.AAQDILLAR.A |
| AtC 6232 | fructose-bisphosphatealdolase [*A.thaliana*] | gi\|15231715 | 96 | 38858/6.05 | 16 | 4 |  | K.AAQEALYVR.C  K.VSPEVIAEHTVR.A  K.GILAADESTGTIGKR.L  R.TVPAAVPAIVFLSGGQSEEEATR.N |
| AtC 6529 | fructose-bisphosphatealdolase [*A.thaliana*] | [gi\|15231715](http://www.matrixscience.com/cgi/protein_view.pl?file=..%2Fdata%2F20140222%2FFTnplbYSR.dat&hit=gi%7C15231715&db_idx=1&px=1&ave_thresh=52&_ignoreionsscorebelow=0&report=0&_sigthreshold=0.05&_msresflags=1025&_msresflags2=2&percolate=0&percolate_rt=0&_minpeplen=7&sessionID=guest_guestsession) | 72 | 38858/6.05 | 5 | 2 |  | K.AAQEALYVR.C  K.VSPEVIAEHTVR.A |
| AtC 7428 | fructose-bisphosphatealdolase [*A.thaliana*] | gi\|15231715 | 357 | 38858 /6.05 | 31 | 7 |  | K.AAQEALYVR.C  K.VSPEVIAEHTVR.A  K.GILAADESTGTIGKR.L  R.LASINVENVETNRR.N  K.FADELIANAAYIGTPGK.G  K.GTVELAGTDGETTTQGLDGLGDR.C  R.TVPAAVPAIVFLSGGQSEEEATR.N |
| AtC 1107 | ferredoxin-NADP(+)-oxidoreductase 1 [*A.thaliana*] | [gi\|15239282](http://www.matrixscience.com/cgi/protein_view.pl?file=..%2Fdata%2F20140116%2FFTnmSzcSm.dat&hit=gi%7C15239282&db_idx=1&px=1&ave_thresh=44&_ignoreionsscorebelow=0&report=5&_sigthreshold=0.05&_msresflags=1025&_msresflags2=2&percolate=-1&percolate_rt=0&_minpeplen=7&sessionID=guest_guestsession) | 97 | 40643/8.32 | 13 | 3 |  | K.RSEQWNVEVY.-  R.LYSIASSAIGDFGDSK.T  K.ITGDDAPGETWHIVFTTEGEVPYR.E |
| AtC 7418 | Contains similarity to ferredoxin-NADP+ reductase from *A.thaliana* | [gi\|8778996](http://www.matrixscience.com/cgi/protein_view.pl?file=..%2Fdata%2F20140619%2FFTnrSiewh.dat&hit=gi%7C8778996&db_idx=1&px=1&ave_thresh=52&_ignoreionsscorebelow=0&report=0&_sigthreshold=0.05&_msresflags=1025&_msresflags2=2&percolate=-1&percolate_rt=0&_minpeplen=7&sessionID=guest_guestsession) | 53 | 39147/8.65 | 5 | 2 |  | K.KNEEGVIVNR.Y  K.KAEQWNVEVY.- |
| AtC 8315 | Contains similarity to ferredoxin-NADP+ reductase from *A.thaliana* | gi\|8778996 | 41 | 39147/8.65 | 8 | 3 |  | K.KNEEGVIVNR.Y  R.YRPKEPYTGK.C  K.KAEQWNVEVY.- |
| AtC 8232 | formate dehydrogenase [*A.thaliana*] | [gi\|15241492](http://www.matrixscience.com/cgi/protein_view.pl?file=..%2Fdata%2F20140123%2FFTnmIfESO.dat&hit=gi%7C15241492&db_idx=1&px=1&ave_thresh=52&_ignoreionsscorebelow=0&report=5&_sigthreshold=0.05&_msresflags=1025&_msresflags2=2&percolate=-1&percolate_rt=0&_minpeplen=7&sessionID=guest_guestsession) | 309 | 42668/7.12 | 16 | 8 |  | K.GVLIVNNAR.G  K.DGELAPQYR.-  K.KGVLIVNNAR.G  K.GEWNVAGIAYR.A  R.YAAGTKDMLER.Y  K.FVEDLNEMLPK.C  K.FVEDLNEMLPK.C + Oxidation (M  K.CDVIVINMPLTEK.T |
| AtC 6615 | UTP--glucose-1-phosphate uridylyltransferase 1 [*A.thaliana*] | [gi\|15237947](http://www.matrixscience.com/cgi/protein_view.pl?file=..%2Fdata%2F20140221%2FFTnpliHET.dat&hit=gi%7C15237947&db_idx=1&px=1&ave_thresh=52&_ignoreionsscorebelow=0&report=0&_sigthreshold=0.05&_msresflags=1025&_msresflags2=2&percolate=-1&percolate_rt=0&_minpeplen=7&sessionID=guest_guestsession) | 171 | 53971/5.66 | 7 | 3 |  | K.SGFINLVSR.Y  K.VLQLETAAGAAIR.F  R.FFDNAIGVNVPR.S |
| AtC 6616 | UTP--glucose-1-phosphate uridylyltransferase 1 [*A.thaliana*] | [gi\|15237947](http://www.matrixscience.com/cgi/protein_view.pl?file=..%2Fdata%2F20140221%2FFTnpliHET.dat&hit=gi%7C15237947&db_idx=1&px=1&ave_thresh=52&_ignoreionsscorebelow=0&report=0&_sigthreshold=0.05&_msresflags=1025&_msresflags2=2&percolate=-1&percolate_rt=0&_minpeplen=7&sessionID=guest_guestsession) | 171 | 52058/5.73 | 7 | 3 |  | K.SGFINLVSR.Y  K.VLQLETAAGAAIR.F  R.FFDNAIGVNVPR.S |
| AtC 6421 | malate dehydrogenase [*A.thaliana*] | gi\|15219721 | 309 | 35548 /6.11 | 25 | 6 |  | R.ALGQISER.L  K.VQTSSGEKPVR.E  K.MELIDAAFPLLK.G  K.VLVVANPANTNALILK.E  R.NGDWSIVQGLPIDEVSR.K  R.VLVTGAAGQIGYALVPMIAR.G |
| AtC 6427 | Malate dehydrogenase, mitochondrial; Flags: Precursor | [gi\|2497857](http://www.matrixscience.com/cgi/protein_view.pl?file=..%2Fdata%2F20140222%2FFTnplbYSe.dat&hit=gi%7C2497857&db_idx=1&px=1&ave_thresh=65&_ignoreionsscorebelow=0&report=0&_sigthreshold=0.05&_msresflags=1025&_msresflags2=2&percolate=-1&percolate_rt=0&_minpeplen=7&sessionID=guest_guestsession) | 138 | 35860/8.81 | 8 | 2 |  | R.DDLFNINAGIVK.N  K.ALEGADLVIIPAGVPR.K |
| AtC 8432 | malate dehydrogenase [*A.thaliana*] | gi\|15219721 | 197 | 35890/6.11 | 40 | 10 |  | R.VLVTGAAGQIGYALVPMIAR.G  K.MELIDAAFPLLK.G  K.MELIDAAFPLLK.G + Oxidation (M)  K.GVVATTDAVEGCTGVNVAVMVGGFPR.K  K.VLVVANPANTNALILK.E  K.EFAPSIPEKNISCLTR.L  R.ALGQISER.L  R.LSVPVSDVK.N  K.VQTSSGEKPVR.E  R.NGDWSIVQGLPIDEVSR.K |
| AtC 7114 | hydroxypyruvatereductase[*A.thaliana*] | gi\|2055273 | 73 | 42501/7.10 | 7 | 3 |  | K.GQTVGVIGAGR.I  K.EAILVNCSR.G  R.IVEADEFMR.G |
| AtC 2722 | phosphoglycerate kinase, partial [*A.thaliana*] | gi\|1022805 | 127 | 41994/4.93 | 6 | 2 |  | K.FSLAPLVPR.L  R.ADLNVPLDDNQTITDDTR.I |
| AtC 2723 | phosphoglycerate kinase, partial [*A.thaliana*] | [gi\|1022805](http://www.matrixscience.com/cgi/protein_view.pl?file=..%2Fdata%2F20140423%2FFTnTIbYae.dat&hit=gi%7C1022805&db_idx=1&px=1&ave_thresh=65&_ignoreionsscorebelow=0&report=0&_sigthreshold=0.05&_msresflags=1025&_msresflags2=2&percolate=-1&percolate_rt=0&_minpeplen=7&sessionID=guest_guestsession) | 185 | 41994/4.93 | 6 | 2 |  | K.FSLAPLVPR.L  R.ADLNVPLDDNQTITDDTR.I |
| AtC 2403 | carbonic anhydrase 2 [*A.thaliana*] | gi\|30685030 | 117 | 37105/7.06 | 12 | 3 |  | K.YMVFACSDSR.V  K.VLAESESSAFEDQCGR.C  R.EAVNVSLANLLTYPFVR.E |
| AtC 2519 | carbonic anhydrase [*A.thaliana*] | gi\|438449 | 62 | 28686/5.64 | 6 | 1 |  | K.VLAESESSAFEDQCGR.C |
| AtC 3301 | carbonic anhydrase 2 [*A.thaliana*] | gi\|42573371 | 148 | 28668/5.36 | 22 | 4 |  | K.YMVFACSDSR.V  K.EKYETNPALYGELAK.G  K.VLAESESSAFEDQCGR.C  R.EAVNVSLANLLTYPFVR.E |
| AtC 4307 | carbonic anhydrase [*A.thaliana*] | gi\|438449 | 146 | 28686/5.64 | 16 | 3 |  | K.YMVFACSDSR.V  R.VCPSHVLDFHPGDAFVVR.N  K.VLAESESSAFEDQCGR.C |
| AtC 5315 | carbonic anhydrase 1 [*A.thaliana*] | gi\|30678347 | 366 | 29827/5.54 | 21 | 6 |  | K.GGYYDFVK.G  K.AFDPVETIK.Q  K.YMVFACSDSR.V  K.YETNPALYGELAK.G  K.EKYETNPALYGELAK.G  K.VISELGDSAFEDQCGR.C |
| AtC 1222 | chlorophyll a/b binding protein (LHCP AB 180) [*A. thaliana*] | gi\|16374 | 51 | 24979/5.12 | 5 | 1 |  | K.GPSGSPWYGSDR.V |
| AtC 2224 | chlorophyll a/b binding protein (LHCP AB 180) [*A. thaliana*] | gi\|16374 | 71 | 25036/5.12 | 5 | 1 |  | U K.GPSGSPWYGSDR.V |
| AtC 5228 | TPA: putative protein kinase superfamily protein [*Zea mays*] | [gi\|293331757](http://www.matrixscience.com/cgi/protein_view.pl?file=..%2Fdata%2F20140221%2FFTnplzuET.dat&hit=gi%7C293331757&db_idx=1&px=1&ave_thresh=51&_ignoreionsscorebelow=0&report=5&_sigthreshold=0.05&_msresflags=1025&_msresflags2=2&percolate=-1&percolate_rt=0&_minpeplen=7&sessionID=guest_guestsession) | 49 | 65710/6.05 | 1 | 1 |  | R.GDKHYVPSR.S |
| AtC 2114 | aluminum-activated, malate transporter 12 [*A.thaliana*] | gi\|15236718 | 49 | 62898/8.49 | 3 | 1 |  | R.AIFIGTAVFIIGAAATYIR.F |
| AtC 3015 | Full=Putative aconitatehydratase, cytoplasmic; Short=Aconitase; AltName: Full=Citrate hydro-lyase | [gi\|75225211](http://www.matrixscience.com/cgi/protein_view.pl?file=..%2Fdata%2F20140129%2FFTnmOacth.dat&hit=gi%7C75225211&db_idx=1&px=1&ave_thresh=46&_ignoreionsscorebelow=0&report=5&_sigthreshold=0.05&_msresflags=1025&_msresflags2=2&percolate=-1&percolate_rt=0&_minpeplen=7&sessionID=guest_guestsession) | 231 | 98591/5.67 | 13 | 12 |  | R.ILLESAIR.N  K.DFNSYGSR.R  K.DGKEVFFR.D  K.LYVFDAALK.Y  R.IDKLPYSIR.I  K.LAEIPFKPAR.V  K.FVEFYGEGMGK.L  K.FYSLPALNDPR.I  R.SDETVAMIEAYLR.A  K.MFVDYNEPQTER.V  K.MFVDYNEPQTER.V + Oxidation (M)  R.VLLQDFTGVPAVVDLAAMR.D |
| AtC 5126 | glutathione S-transferase [*A.thaliana*] | [gi\|11095988](http://www.matrixscience.com/cgi/protein_view.pl?file=..%2Fdata%2F20140123%2FFTnmIfTmt.dat&hit=gi%7C11095988&db_idx=1&px=1&ave_thresh=52&_ignoreionsscorebelow=0&report=5&_sigthreshold=0.05&_msresflags=1025&_msresflags2=2&percolate=-1&percolate_rt=0&_minpeplen=7&sessionID=guest_guestsession) | 108 | 23539/6.31 | 17 | 4 |  | R.VLIALHEK.N  K.VLDVYEHR.L  K.VPAFEDGDFK.L  K.VFGHPASTATR.R |
| AtC 3017 | AT4G02520 [*A.thaliana*]  glutathione S-transferaseF2 [*A.thaliana*] | [gi\|227206270](http://www.matrixscience.com/cgi/protein_view.pl?file=..%2Fdata%2F20140129%2FFTnmOauaT.dat&hit=gi%7C227206270&db_idx=1&px=1&ave_thresh=44&_ignoreionsscorebelow=0&report=5&_sigthreshold=0.05&_msresflags=1025&_msresflags2=2&percolate=-1&percolate_rt=0&_minpeplen=7&sessionID=guest_guestsession)  ref\|NP_192161.1\| | 45 | 12875/5.38 | 29 | 3 |  | K.VLDVYEAR.L  K.LAFEQIFK.S  M.AIGMQVEDHQFDPVASK.L |
| AtC 6226 | F3F9.11 [*A.thaliana*]  glutathione S-transferase TAU 21 [*A.thaliana*] | gi\|8052534  ref\|NP_177957.1\| | 96 | 77520/5.65 | 5 | 3 |  | R.FWADFIDKK.L  K.GVEFEYREEDLR.N  K.SLPDPEKVTEFVSELR.K |
| AtC 5125 | Chain A, Structure Of Glutathione S-Transferase | [gi\|2554769](http://www.matrixscience.com/cgi/protein_view.pl?file=..%2Fdata%2F20140123%2FFTnmIfEOL.dat&hit=gi%7C2554769&db_idx=1&px=1&ave_thresh=52&_ignoreionsscorebelow=0&report=5&_sigthreshold=0.05&_msresflags=1025&_msresflags2=2&percolate=-1&percolate_rt=0&_minpeplen=7&sessionID=guest_guestsession) | 93 | 23983/5.93 | 10 | 3 |  | K.LFTERPR.V  K.VLDVYEAR.L  K.LAFEQIFK.S |
| AtC 5229 | Chain A, Structure Of Glutathione S-Transferase | [gi\|2554769](http://www.matrixscience.com/cgi/protein_view.pl?file=..%2Fdata%2F20140129%2FFTnmOrSwT.dat&hit=gi%7C2554769&db_idx=1&px=1&ave_thresh=46&_ignoreionsscorebelow=0&report=5&_sigthreshold=0.05&_msresflags=1025&_msresflags2=2&percolate=-1&percolate_rt=0&_minpeplen=7&sessionID=guest_guestsession) | 212 | 23983/5.93 | 22 | 5 |  | K.VLDVYEAR.L  R.AITQYIAHR.Y  K.VFGHPASIATR.R  R.NPFGQVPAFEDGDLK.L  R.NPFGQVPAFEDGDLKLFESR.A |
| AtC 7336 | Chain A, Structure Of Glutathione S-Transferase | [gi\|2554769](http://www.matrixscience.com/cgi/protein_view.pl?file=..%2Fdata%2F20140827%2FFTgAizTeO.dat&hit=gi%7C2554769&db_idx=1&px=1&ave_thresh=52&_ignoreionsscorebelow=0&report=0&_sigthreshold=0.05&_msresflags=1025&_msresflags2=2&percolate=-1&percolate_rt=0&_minpeplen=7&sessionID=guest_guestsession) | 158 | 23983/5.98 | 28 | 5 |  | K.VLDVYEAR.L  R.AITQYIAHR.Y  K.VFGHPASIATR.R  R.NPFGQVPAFEDGDLK.L  K.SIYGLTTDEAVVAEEEAK.L |
| AtC 8051 | Chain A, Structure Of Glutathione S-Transferase | [gi\|2554769](http://www.matrixscience.com/cgi/protein_view.pl?file=..%2Fdata%2F20140221%2FFTnplxsmS.dat&hit=gi%7C2554769&db_idx=1&px=1&ave_thresh=52&_ignoreionsscorebelow=0&report=5&_sigthreshold=0.05&_msresflags=1025&_msresflags2=2&percolate=0&percolate_rt=0&_minpeplen=7&sessionID=guest_guestsession) | 75 | 23983/5.93 | 13 | 2 |  | R.YENQGTNLLQTDSK.N  R.NPFGQVPAFEDGDLK.L |
| AtC 1116 | Full=2-CysperoxiredoxinBAS1, chloroplastic | gi\|3121825 | 212 | 29049/7.70 | 20 | 4 |  | K.LSDYIGKK.Y  K.SFGVLIHDQGIALR.G  K.SGGLGDLNYPLISDVTK.S  K.APDFEAEAVFDQEFIK.V |
| AtC 1233 | 2-Cysperoxiredoxinbas1 [*A.thaliana*] | gi\|1498198 | 34 | 28966/8.76 | 5 | 1 |  | K.SFGVLIHDQGIALR.G |
| AtC 6639 | monodehydroascorbatereductase [*A.thaliana*] | gi\|9081770 | 189 | 53528/8.11 | 10 | 4 |  | R.VFEYEGSPR.K  K.YGSLIIATGCTASR.F  K.EAYAPYERPALTK.A  R.EFVIVGGGNAAGYAAR.T |
| AtC 7514 | monodehydroascorbatereductase (NADH) [*A.thaliana*] | [gi\|15231702](http://www.matrixscience.com/cgi/protein_view.pl?file=..%2Fdata%2F20140221%2FFTnpliHSS.dat&hit=gi%7C15231702&db_idx=1&px=1&ave_thresh=51&_ignoreionsscorebelow=0&report=0&_sigthreshold=0.05&_msresflags=1025&_msresflags2=2&percolate=-1&percolate_rt=0&_minpeplen=7&sessionID=guest_guestsession) | 118 | 46629/6.41 | 13 | 4 |  | K.GYLFPEGAAR.L  K.YIILGGGVSAGYAAK.E  K.YQTLIIATGSTVLR.L  K.TSVPDVYAVGDVATFPLK.M |
| AtC 8502 | monodehydroascorbatereductase 1 [*A.thaliana*] | [gi\|15231702](http://www.matrixscience.com/cgi/protein_view.pl?file=..%2Fdata%2F20140812%2FFTgAoxYeh.dat&hit=gi%7C15231702&db_idx=1&px=1&ave_thresh=38&_ignoreionsscorebelow=0&report=0&_sigthreshold=0.05&_msresflags=1089&_msresflags2=2&percolate=-1&percolate_rt=0&_minpeplen=7&sessionID=guest_guestsession) | 39 | 46629/6.41 | 12 | 4 |  | K.GYLFPEGAAR.L  K.ARPSAESLDELVK.Q  K.EAVAPYERPALSK.G  K.AAEGGAAVEEYDYLPFFYSR.S |
| AtC 1112 | 2-Cysperoxiredoxinbas1 [*A.thaliana*] | gi\|1498198 | 115 | 28852/8.76 | 11 | 2 |  | K.SFGVLIHDQGIALR.G  K.SGGLGDLNYPLISDVTK.S |
| AtC 1210 | 2-cysperoxiredoxin-like protein [*A.thaliana*] | [gi\|9758409](http://www.matrixscience.com/cgi/protein_view.pl?file=..%2Fdata%2F20140809%2FFTgAoaumL.dat&hit=gi%7C9758409&db_idx=1&px=1&ave_thresh=64&_ignoreionsscorebelow=0&report=0&_sigthreshold=0.05&_msresflags=1025&_msresflags2=2&percolate=-1&percolate_rt=0&_minpeplen=7&sessionID=guest_guestsession) | 88 | 29543/5.5 | 11 | 2 |  | K.SFGVLIPDQGIALR.G  K.SGGLGDLNYPLVSDITK.S |
| AtC 1238 | 2-Cysperoxiredoxin [*A.thaliana*] | [gi\|7242491](http://www.matrixscience.com/cgi/protein_view.pl?file=..%2Fdata%2F20140423%2FFTnTIbTtT.dat&hit=gi%7C7242491&db_idx=1&px=1&ave_thresh=39&_ignoreionsscorebelow=0&report=0&_sigthreshold=0.05&_msresflags=1089&_msresflags2=2&percolate=-1&percolate_rt=0&_minpeplen=7&sessionID=guest_guestsession) | 42 | 29230/7.74 | 12 | 2 |  | K.SGGLGDLNYPLISDVTK.S  K.APDFEAEAVFDQEFIK.V |
| AtC 2110 | 2-CysperoxiredoxinBAS1 [*A.thaliana*] | gi\|15229806 | 130 | 29188/6.92 | 17 | 3 |  | K.SFGVLIHDQGIALR.G  K.SGGLGDLNYPLISDVTK.S  K.APDFEAEAVFDQEFIK.V |
| AtC 2335 | 2-Cysperoxiredoxinbas1 [*A.thaliana*] | [gi\|131974](http://www.matrixscience.com/cgi/protein_view.pl?file=..%2Fdata%2F20140221%2FFTnplxuwL.dat&hit=gi%7C131974&db_idx=1&px=1&ave_thresh=64&_ignoreionsscorebelow=0&report=0&_sigthreshold=0.05&_msresflags=1025&_msresflags2=2&percolate=-1&percolate_rt=0&_minpeplen=7&sessionID=guest_guestsession) | 38 | 98243/7.01 | 1 | 1 |  | R.DLGNLVSLR.H |
| AtC 127 | thioredoxinM1 [*A.thaliana*] | gi\|30678634 | 104 | 19880/9.14 | 15 | 2 |  | K.MIDPIVNELAQK.Y  K.LNTDESPATPGQYGVR.S |
| AtC 1028 | thioredoxinm2 [*A.thaliana*] | gi\|6539610 | 53 | 20588/9.35 | 8 | 1 |  | K.LNTDESPNTPGQYGVR.S |
| AtC 2622 | glutamate dehydrogenase 2 [*A.thaliana*] | gi\|14423478 | 110 | 24919/7.77 | 14 | 3 |  | R.DLSLSELER.L  K.TAVADIPYGGAK.G  K.DDGTLVSYIGFR.V |
| AtC 6322 | putative disease resistance protein RGA3-like [*Solanumtuberosum*] | gi\|565367052 | 164 | 52139/6.23 | 9 | 5 |  | R.AVYECLR.G  K.DTDILAAFR.V  R.VALEACVKAR.N  K.DDENVNSQPFMR.W  R.GGLDFTKDDENVNSQPFMR.W |
| AtC 1630 | putative dnaK-type molecular chaperone hsc70.1 [*Prunusdulcis*] | [gi\|148807150](http://www.matrixscience.com/cgi/protein_view.pl?file=..%2Fdata%2F20140221%2FFTnplzuOR.dat&hit=gi%7C148807150&db_idx=1&px=1&ave_thresh=52&_ignoreionsscorebelow=0&report=5&_sigthreshold=0.05&_msresflags=1025&_msresflags2=2&percolate=0&percolate_rt=0&_minpeplen=7&sessionID=guest_guestsession) | 50 | 20226/5.07 | 8 | 1 |  | -.SDNQPGVLIQVYEGER.T |
| AtC 3528 | heat shock protein 70 [*A.thaliana*] | [gi\|6746592](http://www.matrixscience.com/cgi/protein_view.pl?file=..%2Fdata%2F20140129%2FFTnmOacTt.dat&hit=gi%7C6746592&db_idx=1&px=1&ave_thresh=46&_ignoreionsscorebelow=0&report=5&_sigthreshold=0.05&_msresflags=1025&_msresflags2=2&percolate=-1&percolate_rt=0&_minpeplen=7&sessionID=guest_guestsession) | 156 | 77230/5.13 | 6 | 4 |  | R.IAGLEVLR.I  K.DIDEVILVGGSTR.I  K.QFAAEEISAQVLR.K  K.AVITVPAYFNDSQR.T |
| AtC 2437 | N-glyceraldehyde-2-phosphotransferase-like [*A.thaliana*] | [gi\|8885622](http://www.matrixscience.com/cgi/protein_view.pl?file=..%2Fdata%2F20140221%2FFTnplzuTh.dat&hit=gi%7C8885622&db_idx=1&px=1&ave_thresh=52&_ignoreionsscorebelow=0&report=5&_sigthreshold=0.05&_msresflags=1025&_msresflags2=2&percolate=0&percolate_rt=0&_minpeplen=7&sessionID=guest_guestsession) | 141 | 31998/5.14 | 8 | 2 |  | R.ENPGCLFIATNR.D  K.LIEGVPETLDMLR.A |
| AtC 2439 | N-glyceraldehyde-2-phosphotransferase-like [*A.thaliana*] | [gi\|8885622](http://www.matrixscience.com/cgi/protein_view.pl?file=..%2Fdata%2F20140221%2FFTnplzuTh.dat&hit=gi%7C8885622&db_idx=1&px=1&ave_thresh=52&_ignoreionsscorebelow=0&report=5&_sigthreshold=0.05&_msresflags=1025&_msresflags2=2&percolate=0&percolate_rt=0&_minpeplen=7&sessionID=guest_guestsession) | 141 | 31998/5.14 | 8 | 2 |  | K.TFQGPPHGIQVER.D  R.ENPGCLFIATNR.D |
| AtC 4413 | alanine aminotransferase-like protein [*A.thaliana*] | [gi\|14596229](http://www.matrixscience.com/cgi/protein_view.pl?file=..%2Fdata%2F20140912%2FFTgAOeTTT.dat&hit=gi%7C14596229&db_idx=1&px=1&ave_thresh=38&_ignoreionsscorebelow=0&report=0&_sigthreshold=0.05&_msresflags=1089&_msresflags2=2&percolate=-1&percolate_rt=0&_minpeplen=7&sessionID=guest_guestsession) | 40 | 42546/5.52 | 15 | 6 |  | K.EVAEFIQR.R  K.GVMQILNCVIR.G  K.GYWGECGQR.G  R.GGYFEMTNLPPR.V  R.GGYFEMTNLPPR.V + Oxidation (M)  K.NVVCNFTEGAMYSFPQIR.L |
| AtC 7629 | putative alanine aminotransferase [*A.thaliana*] | gi\|13430566 | 209 | 53776/6.91 | 10 | 2 |  | K.LNYYTPEYETK.D  K.DDENVNSQPFMR.W |
| AtC 8620 | putative alanine aminotransferase [*A.thaliana*] | gi\|13430566 | 78 | 53776/6.91 | 9 | 4 |  | K.EVAEFIQR.R  K.GYWGECGQR.G  R.GGYFEMTNLPPR.V  K.LLEATGISTVPGSGFGQK.E  K.KNEEGVIVNR.Y  R.YRPKEPYTGK.C  K.KAEQWNVEVY.- |
| AtC 7711 | "5-methyltetrahydropteroyltriglutamate--homocysteinemethyltransferase [*A.thaliana*]  " | gi\|15238686 | 307 | 84646/6.09 | 15 | 8 |  | K.YLFAGVVDGR.N  K.GVTAFGFDLVR.G  K.GGIGVIQIDEAALR.E  R.IPSSEEIADRVNK.M  K.YGAGIGPGVYDIHSPR.I  R.CVKPPVIYGDVSRPK.A  R.YGYTGGEIGLDVYFSMAR.G  K.ALAGQKDEALFSANAAALASR.R |
| AtC 7715 | "5-methyltetrahydropteroyltriglutamate--homocysteinemethyltransferase[*A.thaliana*]  " | [gi\|15238686](http://www.matrixscience.com/cgi/protein_view.pl?file=..%2Fdata%2F20140116%2FFTnmSzcmL.dat&hit=gi%7C15238686&db_idx=1&px=1&ave_thresh=44&_ignoreionsscorebelow=0&report=5&_sigthreshold=0.05&_msresflags=1025&_msresflags2=2&percolate=0&percolate_rt=0&_minpeplen=7&sessionID=guest_guestsession) | 433 | 84646/6.09 | 16 | 9 |  | K.YLFAGVVDGR.N  K.GVTAFGFDLVR.G  R.SDEKLLSVFR.E  K.GGIGVIQIDEAALR.E  R.IPSSEEIADRVNK.M  K.YGAGIGPGVYDIHSPR.I  R.CVKPPVIYGDVSRPK.A  R.YGYTGGEIGLDVYFSMAR.G  K.ALAGQKDEALFSANAAALASR.R |
| AtC 8049 | 5-methyl-tetrahydropteroyltriglutamate-homocysteinemethyltransferase [*A.thaliana*] | [gi\|15238686](http://www.matrixscience.com/cgi/protein_view.pl?file=..%2Fdata%2F20140222%2FFTnplbYEe.dat&hit=gi%7C15238686&db_idx=1&px=1&ave_thresh=52&_ignoreionsscorebelow=0&report=0&_sigthreshold=0.05&_msresflags=1025&_msresflags2=2&percolate=0&percolate_rt=0&_minpeplen=7&sessionID=guest_guestsession) | 175 | 84646/6.09 | 5 | 4 |  | K.YLFAGVVDGR.N  R.IPSSEEIADR.V  K.GVTAFGFDLVR.G  K.GGIGVIQIDEAALR.E |
| AtC 8701 | putative methionine synthase [*A.thaliana*] | [gi\|14532772](http://www.matrixscience.com/cgi/protein_view.pl?file=..%2Fdata%2F20140222%2FFTnplbYEm.dat&hit=gi%7C14532772&db_idx=1&px=1&ave_thresh=52&_ignoreionsscorebelow=0&report=0&_sigthreshold=0.05&_msresflags=1025&_msresflags2=2&percolate=0&percolate_rt=0&_minpeplen=7&sessionID=guest_guestsession) | 151 | 84900/6.09 | 4 | 3 |  | K.YLFAGVVDGR.N  R.IPSTDEIADR.I  K.GGIGVIQIDEAALR.E |
| AtC 7705 | cobalamine-independent methionine synthase, partial [*Ocimumbasilicum*] | gi\|380293559 | 77 | 17231/4.83 | 16 | 2 |  | R.SDEKLISVFR.E  K.YGAGIGPGVYDIHSPR.I |
| AtC 4235 | peptidyl-prolylcis-trans isomeraseCYP20-3 [*A.thaliana*] | gi\|11762200 | 169 | 28532/8.96 | 16 | 4 |  | R.SDEKLISVFR.E  K.YGAGIGPGVYDIHSPR.I |
| AtC 5231 | peptidyl-prolylcis-trans isomeraseCYP20-3 [*A.thaliana*] | [gi\|11762200](http://www.matrixscience.com/cgi/protein_view.pl?file=..%2Fdata%2F20140123%2FFTnmIfEeL.dat&hit=gi%7C11762200&db_idx=1&px=1&ave_thresh=52&_ignoreionsscorebelow=0&report=5&_sigthreshold=0.05&_msresflags=1025&_msresflags2=2&percolate=-1&percolate_rt=0&_minpeplen=7&sessionID=guest_guestsession) | 128 | 46496/8.96 | 10 | 3 |  | K.FEDENFTLK.H  K.VYFDVEIGGEVAGR.I  K.VTNKVYFDVEIGGEVAGR.I |
| AtC 4301 | thiazole biosynthetic enzyme [*A.thaliana*] | gi\|15239735 | 32 | 36755/5.82 | 9 | 2 |  | K.ALDMNTAEDAIVR.L  R.EVVPGMIVTGMEVAEIDGAPR.M |
| AtC 2223 | methylesterase 2 [*A.thaliana* | gi\|15227863 | 45 | 29647/5.17 | 5 | 1 |  | R.VTALDLAASGIDTTR.S |
| AtC 7226 | Adenosylhomocysteinase | [gi\|417744](http://www.matrixscience.com/cgi/protein_view.pl?file=..%2Fdata%2F20140221%2FFTnplzuSS.dat&hit=gi%7C417744&db_idx=1&px=1&ave_thresh=52&_ignoreionsscorebelow=0&report=5&_sigthreshold=0.05&_msresflags=1025&_msresflags2=2&percolate=-1&percolate_rt=0&_minpeplen=7&sessionID=guest_guestsession) | 38 | 53774/5.60 | 1 | 1 |  | R.HSLPDGLMR.A + Oxidation (M) |
| AtC 7418 | Contains similarity to ferredoxin-NADP+ reductase from *A.thaliana* | [gi\|8778996](http://www.matrixscience.com/cgi/protein_view.pl?file=..%2Fdata%2F20140619%2FFTnrSiewh.dat&hit=gi%7C8778996&db_idx=1&px=1&ave_thresh=52&_ignoreionsscorebelow=0&report=0&_sigthreshold=0.05&_msresflags=1025&_msresflags2=2&percolate=-1&percolate_rt=0&_minpeplen=7&sessionID=guest_guestsession) | 53 | 39147/8.65 | 5 | 2 |  | K.KNEEGVIVNR.Y  K.KAEQWNVEVY.- |
| AtC 6233 | actin depolymerizing factor 3 [*A.thaliana*] | gi\|30697295 | 268 | 16026/5.93 | 35 | 3 |  | R.IFFVAWSPDTAR.V   R.YAIFDFDFVSSEGVPR.S  K.IGEPGQTHEDLAASLPADECR.Y |
| AtC 6235 | actin depolymerizing factor 3 [*A.thaliana*] | [gi\|30697295](http://www.matrixscience.com/cgi/protein_view.pl?file=..%2Fdata%2F20140123%2FFTnmIfTne.dat&hit=gi%7C30697295&db_idx=1&px=1&ave_thresh=52&_ignoreionsscorebelow=0&report=5&_sigthreshold=0.05&_msresflags=1025&_msresflags2=2&percolate=-1&percolate_rt=0&_minpeplen=7&sessionID=guest_guestsession) | 272 | 16026/5.93 | 35 | 3 |  | R.IFFVAWSPDTAR.V  R.YAIFDFDFVSSEGVPR.S  K.IGEPGQTHEDLAASLPADECR.Y |
| AtC 6236 | actin depolymerizing factor 3 [*A.thaliana*] | [gi\|30697295](http://www.matrixscience.com/cgi/protein_view.pl?file=..%2Fdata%2F20140221%2FFTnplzuaS.dat&hit=gi%7C30697295&db_idx=1&px=1&ave_thresh=52&_ignoreionsscorebelow=0&report=5&_sigthreshold=0.05&_msresflags=1025&_msresflags2=2&percolate=-1&percolate_rt=0&_minpeplen=7&sessionID=guest_guestsession) | 268 | 16026/5.93 | 35 | 3 |  | R.IFFVAWSPDTAR.V  R.YAIFDFDFVSSEGVPR.S  K.IGEPGQTHEDLAASLPADECR.Y |
| AtC 8152 | nucleoside diphosphate kinase [*A.thaliana*] | [gi\|16396](http://www.matrixscience.com/cgi/protein_view.pl?file=..%2Fdata%2F20140221%2FFTnplxunt.dat&hit=gi%7C16396&db_idx=1&px=1&ave_thresh=51&_ignoreionsscorebelow=0&report=0&_sigthreshold=0.05&_msresflags=1025&_msresflags2=2&percolate=-1&percolate_rt=0&_minpeplen=7&sessionID=guest_guestsession) | 325 | 16286/7.03 | 32 | 5 |  | R.GDFAIDIGR.N  R.GLIGEVICR.F  R.NVIHGSDSVESAR.K  K.IIGATNPAASEPGTIR.G  R.KIIGATNPAASEPGTIR.G |
| AtC 5124 | Chain A, Crystal Structure Of Nucleoside Diphosphate Kinase 2 From Arabidopsis | [gi\|56965987](http://www.matrixscience.com/cgi/protein_view.pl?file=..%2Fdata%2F20140123%2FFTnmIfTTR.dat&hit=gi%7C56965987&db_idx=1&px=1&ave_thresh=52&_ignoreionsscorebelow=0&report=5&_sigthreshold=0.05&_msresflags=1025&_msresflags2=2&percolate=-1&percolate_rt=0&_minpeplen=7&sessionID=guest_guestsession) | 143 | 17220/5.54 | 41 | 5 |  | R.GDLAVQTGR.N  R.GLVGEIISR.F  K.TDPLQAEPGTIR.G  R.NIVHGSDSPENGKR.E  -.SMEDVEETYIMVKPDGIQR.G |
| AtC 2236 | PREDICTED: E3 ubiquitin-protein ligase At3g02290-like [*Setariaitalica*] | [gi\|527209537](http://www.matrixscience.com/cgi/protein_view.pl?file=..%2Fdata%2F20140221%2FFTnplzumO.dat&hit=gi%7C527209537&db_idx=1&px=1&ave_thresh=52&_ignoreionsscorebelow=0&report=5&_sigthreshold=0.05&_msresflags=1025&_msresflags2=2&percolate=-1&percolate_rt=0&_minpeplen=7&sessionID=guest_guestsession) | 66 | 45703/7.33 | 2 | 1 |  | R.VLMMTKSR.M + Oxidation (M) |
| AtC 7161 | predicted protein [*Bathycoccusprasinos*] eukaryotic translation initiation factor-like protein [*Micromonas* sp. RCC299] | [gi\|412986794](http://www.matrixscience.com/cgi/protein_view.pl?file=..%2Fdata%2F20140123%2FFTnmIfeOt.dat&hit=gi%7C412986794&db_idx=1&px=1&ave_thresh=52&_ignoreionsscorebelow=0&report=5&_sigthreshold=0.05&_msresflags=1025&_msresflags2=2&percolate=-1&percolate_rt=0&_minpeplen=7&sessionID=guest_guestsession)  ref\|XP_002505347.1 | 39 | 83968/5.44 | 1 | 1 |  | R.ISLMQLPSR.N |
| AtC 7326 | rop guanine nucleotide exchange factor 1-like [*Solanumlycopersicum*] | [gi\|460401771](http://www.matrixscience.com/cgi/protein_view.pl?file=..%2Fdata%2F20140221%2FFTnplzunS.dat&hit=gi%7C460401771&db_idx=1&px=1&ave_thresh=52&_ignoreionsscorebelow=0&report=5&_sigthreshold=0.05&_msresflags=1025&_msresflags2=2&percolate=-1&percolate_rt=0&_minpeplen=7&sessionID=guest_guestsession) | 26 | 75266/5.38 | 1 | 1 |  | R.TQAKSSLLR.S |
| AtC 5228 | U-box domain-containing protein 38 [*A.thaliana*] | [gi\|15238366](http://www.matrixscience.com/cgi/protein_view.pl?file=..%2Fdata%2F20140221%2FFTnplzsmL.dat&hit=gi%7C15238366&db_idx=1&px=1&ave_thresh=52&_ignoreionsscorebelow=0&report=5&_sigthreshold=0.05&_msresflags=1025&_msresflags2=2&percolate=-1&percolate_rt=0&_minpeplen=7&sessionID=guest_guestsession) | 26 | 62310/6.24 | 2 | 1 |  | R.APMIIHHADSELMGR.R + Oxidation (M) |
| AtC 8057 | Uncharacterized RNA-binding protein C25G10.01 [*Triticumurartu*] | [gi\|474216345](http://www.matrixscience.com/cgi/protein_view.pl?file=..%2Fdata%2F20140129%2FFTnmOaant.dat&hit=gi%7C474216345&db_idx=1&px=1&ave_thresh=46&_ignoreionsscorebelow=0&report=5&_sigthreshold=0.05&_msresflags=1025&_msresflags2=2&percolate=-1&percolate_rt=0&_minpeplen=7&sessionID=guest_guestsession) | 38 | 74448/6.71 | 1 | 1 |  | R.DDEMAER.W + Oxidation (M) |
| AtC 1027 | probable inactive receptor kinase At1g48480 [*Vitisvinifera*] | [gi\|225424347](http://www.matrixscience.com/cgi/protein_view.pl?file=..%2Fdata%2F20140129%2FFTnmOauth.dat&hit=gi%7C225424347&db_idx=1&px=1&ave_thresh=46&_ignoreionsscorebelow=0&report=5&_sigthreshold=0.05&_msresflags=1025&_msresflags2=2&percolate=-1&percolate_rt=0&_minpeplen=7&sessionID=guest_guestsession) | 25 | 67050/5.66 | 1 | 1 |  | R.LSGSIPKGLR.N |
| AtC 2420 | adenosine kinase 1 [*A.thaliana*] | [gi\|15232763](http://www.matrixscience.com/cgi/protein_view.pl?file=..%2Fdata%2F20140827%2FFTgAizTTE.dat&hit=gi%7C15232763&db_idx=1&px=1&ave_thresh=52&_ignoreionsscorebelow=0&report=0&_sigthreshold=0.05&_msresflags=1025&_msresflags2=2&percolate=-1&percolate_rt=0&_minpeplen=7&sessionID=guest_guestsession) | 67 | 38268/5.29 | 8 | 2 |  | K.FNVEYIAGGATQNSIK.V  R.AGCYASNVVIQR.S |
| AtC 2024 | F1O19.10/F1O19.10 [*Arabidopsis thaliana*] | gi\|13926229 | 304 | 14917/5.69 | 52 | 6 |  | K.EVDYLIR.N  R.EHGNSPGYYDGR.Y  K.LPLFGCTDSAQVLK.E  K.EYPNAFIR.I  R.IIGFDNTR.Q  R.QVQCISFIAYKPPSFTG.- |
| AtC 3015 | F1O19.10/F1O19.10 [*Arabidopsis thaliana*] | gi\|13926229 | 301 | 14917/5.69 | 52 | 6 | \|  \| \| --- \| \|  \| | K.EVDYLIR.N  K.EYPNAFIR.I  R.EHGNSPGYYDGR.Y  K.LPLFGCTDSAQVLK.E  R.QVQCISFIAYKPPSFTG.- |
| AtC 8430 | F1O19.10/F1O19.10 [*A.thaliana*] | gi\|13926229 | 55 | 14917/5.69 | 18 | 3 |  | K.EVDYLIR.N  K.EYPNAFIR.I  R.IIGFDNTR.Q |
| AtC 8529 | F1O19.10/F1O19.10 [*A.thaliana*] | gi\|13926229 | 55 | 14689/5.69 | 18 | 3 |  | K.EVDYLIR.N  R.IIGFDNTR.Q  K.EYPNAFIR.I |
| AtC 1030 | hypothetical protein CARUB_v10005847mg [*Capsella rubella*] | [gi\|565446290](http://www.matrixscience.com/cgi/protein_view.pl?file=..%2Fdata%2F20140809%2FFTgAoaatO.dat&hit=gi%7C565446290&db_idx=1&px=1&ave_thresh=51&_ignoreionsscorebelow=0&report=0&_sigthreshold=0.05&_msresflags=1025&_msresflags2=2&percolate=-1&percolate_rt=0&_minpeplen=7&sessionID=guest_guestsession) | 125 | 20500/7.57 | 12 | 3 |  | K.EVDYLLR.N  R.IIGFDNTR.Q  K.EYPNAFIR.I |
| AtC 2235 | hypothetical protein [*Oryzasativa* Japonica Group] | [gi\|42408418](http://www.matrixscience.com/cgi/protein_view.pl?file=..%2Fdata%2F20140221%2FFTnplxsEh.dat&hit=gi%7C42408418&db_idx=1&px=1&ave_thresh=53&_ignoreionsscorebelow=0&report=5&_sigthreshold=0.05&_msresflags=1025&_msresflags2=2&percolate=-1&percolate_rt=0&_minpeplen=7&sessionID=guest_guestsession) | 42 | 16945/11.15 | 5 | 1 |  | -.MAGEKGAAR.G |
| AtC 4229 | hypothetical protein GLOTRDRAFT_65728 [*Gloeophyllumtrabeum*ATCC 11539] |  | 115 |  | 3 | 1 |  | R.QYESQIAELEKR.L |
| AtC 6230 | hypothetical protein VOLCADRAFT_97820 [*Volvoxcarteri f. nagariensis*] | [gi\|302850507](http://www.matrixscience.com/cgi/protein_view.pl?file=..%2Fdata%2F20140221%2FFTnplxsOT.dat&hit=gi%7C302850507&db_idx=1&px=1&ave_thresh=52&_ignoreionsscorebelow=0&report=5&_sigthreshold=0.05&_msresflags=1025&_msresflags2=2&percolate=-1&percolate_rt=0&_minpeplen=7&sessionID=guest_guestsession) | 19 | 22397/8.16 | 4 | 1 |  | M.SLRPQKFVR.D |
| AtC 7415 | hypothetical protein PHAVU_001G054400g [*Phaseolusvulgaris*] | [gi\|561034711](http://www.matrixscience.com/cgi/protein_view.pl?file=..%2Fdata%2F20140221%2FFTnplzuTO.dat&hit=gi%7C561034711&db_idx=1&px=1&ave_thresh=51&_ignoreionsscorebelow=0&report=5&_sigthreshold=0.05&_msresflags=1025&_msresflags2=2&percolate=0&percolate_rt=0&_minpeplen=7&sessionID=guest_guestsession) | 33 | 164113/8.44 | 1 | 1 |  | -.MEDSGISRVDSAR.G + Oxidation (M) |
| AtC 8411 | hypothetical protein SORBIDRAFT_07g021965 [*Sorghum bicolor*] | gi\|242081629 | 42 | 90955/8.56 | 1 | 1 |  | R.ITEAVADLR.F |
| AtC 4236 | AT3g62030 [*A.thaliana*] | gi\|11762200 | 149 | 28532/8.96 | 14 | 4 |  | K.FEDENFTLK.H  K.VYFDVEIGGEVAGR.I  K.VTNKVYFDVEIGGEVAGR.I |
| AtC 6619 | AT3g03250/T17B22_6 [*A.thaliana*] | gi\|13605559 | 111 | 51847/5.93 | 7 | 3 |  | K.SGFISLVSR.Y   K.VLQLETAAGAAIR.F   R.FFDNAIGVNVPR.S |
| AtC 6018 | unnamed protein product [*Hordeumvulgare*] | [gi\|11587](http://www.matrixscience.com/cgi/protein_view.pl?file=..%2Fdata%2F20140913%2FFTgcfrsaR.dat&hit=gi%7C11587&db_idx=1&px=1&ave_thresh=54&_ignoreionsscorebelow=0&report=5&_sigthreshold=0.05&_msresflags=1025&_msresflags2=2&percolate=-1&percolate_rt=0&_minpeplen=7&sessionID=guest_guestsession) | 54 | 47517/6.32 | 3 | 1 |  | K.TFQGPPHGIQVER.D |
| AtC 129 | MD-2-related lipid recognition domain-containing protein [*A.thaliana*] | [gi\|15237836](http://www.matrixscience.com/cgi/protein_view.pl?file=..%2Fdata%2F20140827%2FFTgAizTSS.dat&hit=gi%7C15237836&db_idx=1&px=1&ave_thresh=53&_ignoreionsscorebelow=0&report=0&_sigthreshold=0.05&_msresflags=1025&_msresflags2=2&percolate=-1&percolate_rt=0&_minpeplen=7&sessionID=guest_guestsession) | 69 | 18124/4.57 | 13 | 2 |  | R.DGEFTGLLK.T   K.NIPAGTLVYVAFR.D |
