# Supplementary material for: Omics-Based Mechanistic Insight Into the Role of Bioengineered Nanoparticles for Biotic Stress Amelioration by Modulating Plant Metabolic Pathways
Source: Front Bioeng Biotechnol. 2020 Apr 17;8:242. doi: 10.3389/fbioe.2020.00242 (PMC7180193; doi:10.3389/fbioe.2020.00242)
Supplement: Supplementary file 5 [file Table_4.DOCX]

**Table S4** Putative domain analysis of miscellaneous proteins differentially expressed in plant pathogen nanoparticles interaction

| **Spot ID** | **Protein identification** | **Interpro ID** | **Domain name** |
| --- | --- | --- | --- |
| 3015 | F1O19.10/F1O19.10 [*Arabidopsis* *thaliana*] | IPR024681 | Ribulosebisphosphate carboxylase, small chain |
| 8529 | F1O19.10/F1O19.10 [*Arabidopsis thaliana*] | IPR024681 | Ribulosebisphosphate carboxylase, small chain |
| 2024 | F1O19.10/F1O19.10 [*Arabidopsis thaliana*] | IPR000894 | Ribulosebisphosphate carboxylase, small chain |
| 8431 | F1O19.10/F1O19.10 [*Arabidopsis thaliana*] | IPR000894 | Ribulosebisphosphate carboxylase small chain, domain |
| 1030 | hypothetical protein CARUB_v10005847mg [*Capsella rubella*] | IPR024681 | Ribulosebisphosphate carboxylase, small chain |
| 2235 | hypothetical protein [*Oryza sativa Japonica* Group] |  | Non predicted |
| 4229 | hypothetical protein GLOTRDRAFT_65728 [*Gloeophyllum trabeum* ATCC 11539] | IPR008989 | Myosin S1 fragment, N-terminal |
| 6230 | hypothetical protein VOLCADRAFT_97820 [*Volvox carteri f. nagariensis*] | IPR029063 | S-adenosyl-L-methionine-dependent methyltransferase-like |
| 7415 | hypothetical protein PHAVU_001G054400g [*Phaseolus vulgaris*] | IPR029481 | ABC-transporter extracellular N-terminal domain |
| 8411 | hypothetical protein SORBIDRAFT_07g021965 [*Sorghum bicolor*] | IPR001878 | Zinc finger, CCHC-type |
| 4236 | AT3g62030 [*Arabidopsis thaliana*] | IPR029000 | Cyclophilin-like domain |
| 6619 | AT3g03250/T17B22_6 [*Arabidopsis thaliana*] | IPR029044 | Nucleotide-diphospho-sugar transferases |
